# Taxon‐Specific Loss in Scleractinian Living Coral Area Following a Bleaching Event Is Linked to Reef Structural Decline in the Central Red Sea

**DOI:** 10.1002/ece3.74565

**Published:** 2026-10-30

**Authors:** Natalie Dunn, Fabio Marchese, Marta Ezeta Watts, Megan K. B. Nolan, Francesca Benzoni

**Affiliations:** ^1^ Marine Science Program King Abdullah University of Science and Technology (KAUST) Thuwal Kingdom of Saudi Arabia; ^2^ Biological and Environmental Science and Engineering Division King Abdullah University of Science and Technology (KAUST) Thuwal Kingdom of Saudi Arabia

**Keywords:** climate change, coral bleaching, photogrammetry, Red Sea, structural complexity

## Abstract

Marine heatwaves are increasing in frequency and intensity worldwide, triggering coral bleaching events. While colony and community scale consequences of bleaching have been well documented, its consequence on reef structural complexity remains comparatively less studied. Using Structure‐from‐Motion (SfM), colony fate tracking of hermatypic scleractinians, and a cross‐scale, sequential approach, this study linked in situ thermal stress to coral colony‐scale bleaching and mortality and reef‐scale structural change during the 2023 bleaching event in the central Saudi Arabian Red Sea. Taxon‐specific bleaching thresholds were consistent with regional bleaching alert levels; however, ecological risk varied substantially among reefs. We further support that the relationship between thermal stress and change in reef‐building coral colony area is taxon‐dependent, and that bleaching‐driven coral mortality can rapidly alter reef structural complexity, with potential consequences for ecosystem function. This study represents the first mechanistic assessment of coral resilience across biological scales in the central Red Sea, and provides new insight into reef structural persistence under accelerating climate change.

## Graphical abstract

Coral bleaching is becoming more frequent and severe worldwide, yet its effects on reef structural complexity remain poorly understood compared with the well‐documented impacts on coral colonies and communities. We used a cross‐scale approach to link in situ thermal stress to coral colony‐scale bleaching and mortality and reef‐scale structural change during the 2023 bleaching event in the central Saudi Arabian Red Sea. This study showed that reef‐scale structural vulnerability to heat stress is governed by taxon‐specific responses that may not be captured by regional thermal metrics and represents the first mechanistic assessment of coral resilience across biological scales in the central Red Sea.

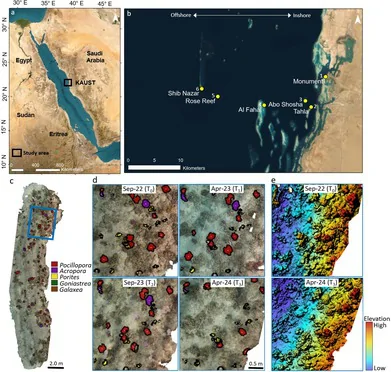

## 1 Introduction

Structural complexity generated by key taxa shapes biodiversity and ecological function across ecosystems by increasing habitat heterogeneity, supporting diverse communities, and modulating species interactions (MacArthur 1984; Ellison et al. 2005; Graham and Nash 2013). In shallow coral reef ecosystems, the coral reef three‐dimensional, complex biogenic structure is formed primarily by hermatypic scleractinian corals that secrete a calcium carbonate skeleton (Luckhurst and Luckhurst 1978; McClanahan 1994; Wilson et al. 2007; Fukunaga, Burns, et al. 2020). The reef framework structure and complexity underpin biodiversity of reef‐associated communities by providing habitat across multiple spatial scales from centimeters to hundreds of meters (Risk 1972; Holbrook et al. 2003; Purkis et al. 2008; Graham and Nash 2013; Bozec et al. 2015; Roff et al. 2020). Coral reef structural complexity also drives key ecosystem processes such as productivity and trophic interactions (Alvarez‐Filip et al. 2011; Darling et al. 2017) and mediates the capacity of their resilience (Graham et al. 2006; Pratchett et al. 2008). Coral reef structure further provides associated ecosystem services such as supporting fisheries (Rogers et al. 2014; Richardson, Graham, Pratchett, and Hoey 2017) and protecting shorelines by dissipating wave energy (Sheppard et al. 2005). However, these ecological functions and ecosystem services are declining with the loss of structural complexity (reef flattening) due to multiple threats (Hoegh‐Guldberg 1999; Wilkinson 2002; Pandolfi et al. 2003; Baker et al. 2008; Alvarez‐Filip 2010; Graham and Nash 2013). This loss triggers cascading effects on ecosystems, with recovery often occurring over decadal timescales (Connell 1997; Riegl 2001; Graham et al. 2007; Gouezo et al. 2019), underscoring the need to incorporate structural complexity in monitoring efforts. For example, physical, ephemeral events such as storms can lead to pronounced and uniform structural degradation (Hughes and Connell 1999; Alvarez‐Filip 2010; Burns et al. 2016; Pascoe et al. 2021), while consequences of chronic disturbances such as sedimentation may drive more subtle or spatially variable structural changes (Rogers 1990; Magel et al. 2019).

Coral reef surface structural complexity has been traditionally described by ecologists by a single, coarse metric such as rugosity estimated by the chain‐and‐tape method (Risk 1972; McCormick 1994; Jokiel et al. 2004; Alvarez‐Filip 2010; Graham and Nash 2013). The calculation of multiple quantitative metrics capturing distinct aspects of structural complexity has only become practical in recent years with the advancement of computer‐generated, high‐resolution, three‐dimensional reconstructions of coral reefs (Burns et al. 2015; Ferrari et al. 2016; Fukunaga, Burns, et al. 2020). Specifically, advances in Structure‐from‐Motion (SfM) approaches now enable precise quantification of various metrics of three‐dimensional complexity across multiple spatial scales, facilitated by the development of standardized and automated protocols for metrics extraction (Aston et al. 2022), revealing that structural change is scale‐ and metric‐dependent and cannot be captured by a single measure (Ferreira et al. 2023). This methodology also enables the precise geo‐referencing of individual colonies across time (Magel et al. 2019; Couch et al. 2021; Ferreira et al. 2023; Kopecky et al. 2023). Indeed, different metrics describe distinct dimensions of reef architecture and have been related to different coral morphologies and ecological functions (Fukunaga, Kosaki, et al. 2020; Fukunaga, Burns, et al. 2020; Lazarus and Belmaker 2021; Helder et al. 2022). For example, at 1 cm resolution, slope has been suggested to best capture overall changes in vertical relief, reflecting large‐scale three‐dimensional structure generated by coral colony size and substratum, while Vector Ruggedness Measure (VRM) has been used to capture small‐scale surface complexity generally created by branching, encrusting corals and rubble, and profile curvature measures local convexity or concavity, best capturing holes and ledges (Fukunaga, Burns, et al. 2020; Pascoe et al. 2021; Helder et al. 2022; Asbury et al. 2023; Ferreira et al. 2023). These complementary dimensions of complexity have been linked to ecological functions ranging from microhabitat availability for cryptic, small‐bodied organisms to hydrodynamic modifications (Lazarus and Belmaker 2021; Helder et al. 2022). Despite this growing understanding, studies have focused on structural change following catastrophic events such as hurricanes (Burns et al. 2016; Pascoe et al. 2021), while the structural consequences of biological disturbances, such as coral bleaching, over short timescales are less understood (Alvarez‐Filip 2010; Couch et al. 2017).

Coral bleaching can occur when elevated thermal stress linked to anthropogenic climate change disrupts the coral‐symbiont association, causing the coral to expel the associated photosynthetic Symbiodiniaceae (zooxanthellae) and appear visibly white (Jokiel and Coles 1990; Baird et al. 2009; Lesser 2010; Baker and Cunning 2015). As these endosymbionts provide the majority of the host's energetic requirements, coral bleaching can lead to reduced growth, starvation, and mortality in zooxanthellate corals (Muscatine et al. 1984; Fitt et al. 1993; Douglas 2003; Baker et al. 2008; Rädecker et al. 2021). Increasingly frequent and intense marine heatwaves are driving widespread coral mortality (Hoegh‐Guldberg 1999; Hughes et al. 2017, 2021; Frölicher and Laufkötter 2018; Eddy et al. 2021), shortening recovery intervals between disturbances and threatening not only coral cover, but the structural integrity of the complex coral framework and overall reef persistence (Hoegh‐Guldberg 1999; Hughes et al. 2017). While coral bleaching and associated declines in coral cover and shifts in colony size have been extensively documented (e.g., Loya et al. 2001; De'Ath et al. 2012; Babcock et al. 2020; Burn et al. 2023; Winslow et al. 2024), fewer studies quantitatively link thermal stress to bleaching responses and subsequent changes in reef structural complexity (Couch et al. 2017; Magel et al. 2019). However, these studies used satellite or categorical heat stress, and taxon‐specific trajectories are critical to predicting how thermal stress translates into functional declines in reef habitat (Matsuda et al. 2020; Fukunaga, Burns, et al. 2020; Ferreira et al. 2023).

Satellite‐derived metrics such as degree heating weeks (DHWs), which quantify cumulative heat stress, are commonly used as drivers to explain bleaching and support regional warning systems such as NOAA Coral Reef Watch alert levels (NOAA CRW 2025). While these tools provide valuable broad‐scale assessments, bleaching responses vary widely among taxa and regions, with branching coral taxa such as *Acropora* generally showing higher susceptibility than massive ones such as *Porites* (e.g., Loya et al. 2001; van Woesik et al. 2011; Grottoli et al. 2014). Thus, spatial variability in thermal regimes and biological susceptibility limit applicability of uniform thresholds from such tools. Additionally, there is limited empirical data linking in situ thermal stress to bleaching severity across taxa. In turn, the bleaching response does not necessarily correspond directly to coral mortality (McClanahan 2004; Baker et al. 2008; Berkelmans 2009; Dunn et al. 2025), and the relationship between the severity of bleaching and change in colony size is lacking. Proxies, such as random points or maximum diameter may be used to annotate the condition of coral colonies (Hill and Wilkinson 2004), however direct measurements of colony surface area would more accurately capture colony size and enable quantification of the sequential empirical links between thermal stress, bleaching response, and mortality at the colony level in order to establish how taxon‐specific responses shape reef structural trajectories (Neal et al. 2015; Ferreira et al. 2023).

The Red Sea, a relatively warm ocean basin with corals living near their thermal maxima (Berumen et al. 2019; Ziegler et al. 2019), offers a natural setting to investigate the mechanistic links between thermal stress and coral responses. In addition, the thermal regime of the marine environment is heterogeneous along cross‐shelf and latitudinal gradients (Sofianos and Johns 2015; Ellis et al. 2017; Berumen et al. 2019), and while it has been suggested that Red Sea corals are thermally tolerant and may exhibit acclimatization (Davis et al. 2011; Sawall et al. 2014; Ziegler et al. 2015), they are increasingly vulnerable under increasing temperatures (Chaidez et al. 2017; Osman et al. 2018; Gonzalez et al. 2024). Indeed, bleaching events have previously been reported in the central Red Sea in 1998 (DeVantier et al. 2000), 2010 (Furby et al. 2013), 2015 (Monroe et al. 2018), 2020 (Dunn et al. 2025), 2023 (Bähr et al. 2025; Tietbohl et al. 2025), and 2024 (Mayfield 2025; Dunn et al. 2026), with generally increasing magnitude. Despite this vulnerability, relatively little is known about in situ thermal regimes, taxon‐specific bleaching thresholds, and reef structural complexity in the central Red Sea, providing an opportunity to elucidate how the consequences of heat stress propagate across these biological scales. Accordingly, the aim of this study was to use an integrated, sequential approach linking in situ thermal stress, coral bleaching, colony‐scale mortality, and reef‐scale changes in structural complexity to quantify the cross‐scale consequences of the 2023 bleaching event in the central Red Sea.

## 2 Materials and Methods

### 2.1 Study Sites

This study was conducted in the central Saudi Arabian Red Sea near King Abdullah University of Science and Technology (KAUST), where 18 permanent transects (three per reef) were installed 5 m to 10 m deep on the sheltered side of 6 reefs spanning a cross‐shelf gradient (Figure 1a,b). Each transect was 5 m wide by 20 m long to align with previous benthic surveys in the region (Ellis et al. 2017). The 6 reefs were (1) Monument (inshore), (2) Tahla (inshore), (3) Abo Shosha (inshore), (4) Al Fahal (midshelf), (5) Rose Reef (offshore), and (6) Shib Nazar (offshore). Reefs were categorized into inshore, midshelf, and offshore according to their relative positions along the cross‐shelf gradient consistent with previous studies in the region (e.g., Furby et al. 2013; Monroe et al. 2018). According to the decision tree of Rowlands et al. (2014) for typology of Red Sea shallow carbonate systems and the bathymetric dataset of Ezeta Watts (2022), Monument (inshore) is a fringing reef with a shallow lagoon, and transects were established on the leeward reef slope, which is characterized by step‐like consolidated reef framework overlaid with fine sediment. Tahla (inshore) is a planar platform system less than 3 km from shore, with transects located at the base of the reef slope, where it transitions to rubble and sandy (unconsolidated) substrate. Abo Shosha (inshore) can be described as a small platform system with lagoonal patches less than 4 km from shore. Transects spanned discrete patch reefs surrounded by sandy seabed (unconsolidated carbonate). Al Fahal (midshelf) is adjacent to deeper waters, and may be characterized as an elongated platform system with lagoonal patch reefs. Transects were established along the leeward slopes of patch reefs at the transition of the base of the slope and the unconsolidated sandy seabed. Rose Reef (offshore) is a planar tower system about 21 km offshore and surrounded by > 200 m deep water. Transects were established along the steep reef wall. Shib Nazar (offshore) is an elongated platform system with lagoonal patches about 24 km offshore. Transects were conducted on the leeward slope and spanned crevice formations in the consolidated carbonate framework.

**FIGURE 1:**
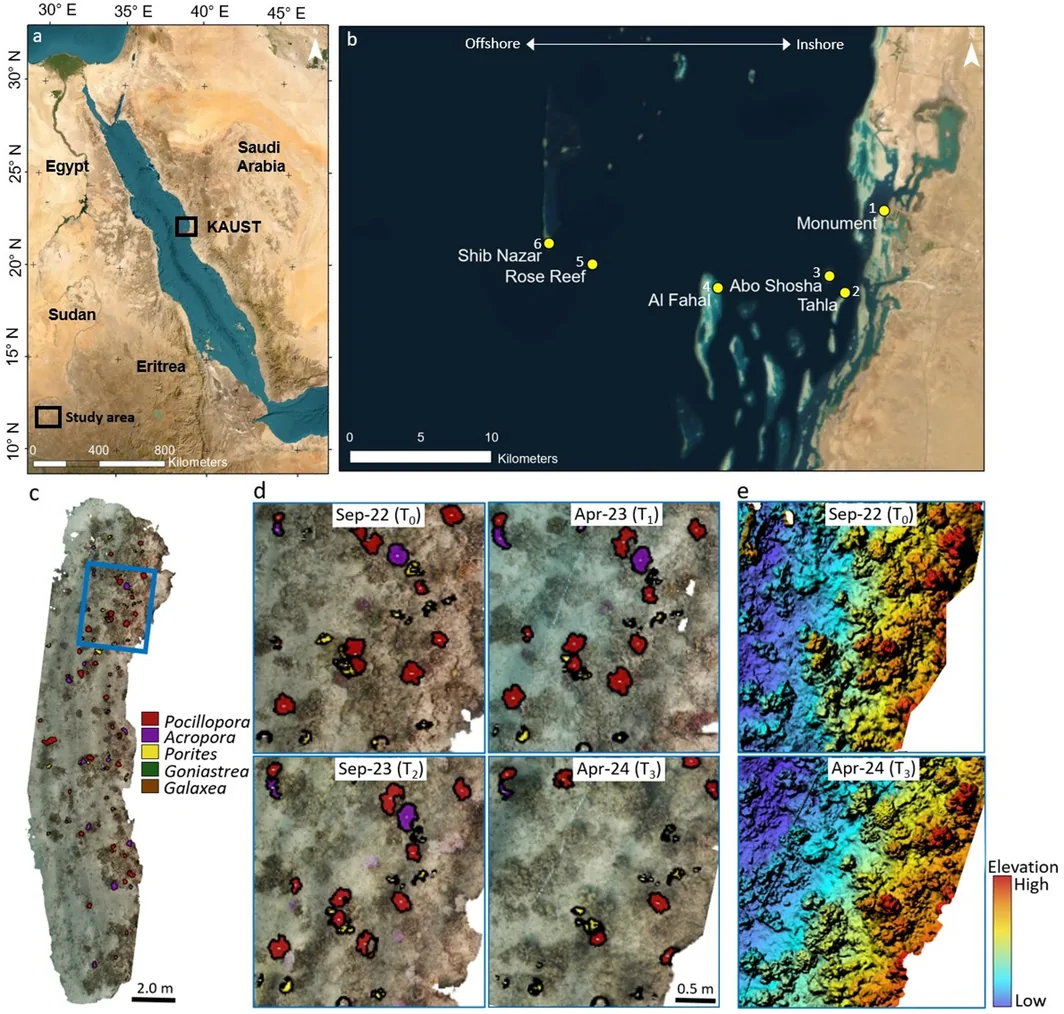
Study sites in the central Saudi Arabian Red Sea. (a) Map of the Red Sea showing the location of the study region (black polygon). (b) Study sites near KAUST in Saudi Arabia surveyed from September 2022 to April 2024. Basemap satellite images accessed from World Imagery ESRI Tile Layer. Credits: Esri, Maxar, GeoEye Earthstar Geographics, CNES/Airbus DS, USDA, USGS, AeroGRID, IGN, and the GIS User Community. (c) Example orthomosaic of one transect on Al Fahal with coral colonies of target taxa annotated in TagLab. (d) Zoom in of orthomosaic with colonies annotated at each survey time point. (e) Digital elevation models of the same zoom in area of the orthomosaic for the first and last time points.

### 2.2 In Situ Temperature

In situ temperature was recorded at 10‐min intervals using Star Oddi DST CT data loggers, highly accurate sensors (Rich et al. 2024), attached to the starting picket of each transect. The in situ daily maximum (DM) temperature was extracted at the transect scale on the day of each survey time point to describe heat exposure for each reef. Degree heating weeks (DHW) were also estimated at the transect scale from the in situ daily minimum on the day of each survey time point using the maximum monthly mean for each reef extracted from the National Oceanic and Atmospheric Administration's (NOAA) Coral Reef Watch (CRW) data. Average DHW and DM for each reef were plotted over time to visualize heat stress for each reef, while DHW, representing a measure of cumulative heat stress at the transect scale, was used in downstream analyses.

### 2.3 Image Acquisition and Model Generation

Imagery was collected following Dunn et al. (2025), whereby a SCUBA diver collected a series of overlapping images 1 m above the substrate. A Sony α7r IV with a 12–24 mm f/2.8 lens set at 24 mm with Easydive Leo3 Wi underwater housing was used for image acquisition. Surveys at *T*_0_ were conducted for Abo Shosha, Al Fahal, Rose Reef, and Shib Nazar in September 2022 (pre‐bleaching) and in November 2022 (pre‐bleaching) for Tahla and Monument. At Tahla at *T*_0_, only two transects were surveyed due to logistical constraints. *T*_1_, *T*_2_, and *T*_3_ surveys were conducted in April 2023 (pre‐bleaching), September 2023 (during bleaching), and April 2024 (post‐bleaching) thereafter for all reefs and transects. An average of about 1500 images per transect was collected at each time point. Images were processed in Agisoft Metashape v2.0 (AgiSoft 2023) to generate three‐dimensional models of each transect using the processing parameters in Dunn et al. (2025) and Ibex, the HPC (High‐Performance Computer) cluster at King Abdullah University of Science and Technology (KAUST, Saudi Arabia). All models were scaled using 25 cm calibrated ground control points, and vertically oriented using a level in the field. The models were decimated to 2.3 million faces to generate 1 cm sized polygons, and digital elevation models (DEMs) were built on decimated models to minimize noise following methodology adapted from Fukunaga and Burns (2020). Orthomosaics and DEMs were exported at 1 mm and 1 cm resolution, respectively, for each transect at each time point for downstream analyzes, where coral colonies were fate tracked using the orthomosaics and metrics of structural complexity were extracted from the DEMs.

### 2.4 Coral Colony Fate Tracking and Structural Complexity Metrics

To characterize benthic communities at *T*_0_, orthomosaics were input into ArcGIS Desktop 10.8.2 and the point‐intercept method was digitally used to identify benthic cover every 0.5 m (Hill and Wilkinson 2004; Urbina‐Barreto et al. 2021). Corals were identified to the lowest possible taxonomic level (typically genus) and non‐coral taxa were identified to functional group following Obura (2014) and Khalil et al. (2017).

The most abundant scleractinian coral genera (*Acropora*, *Pocillopora*, *Porites*, and *Goniastrea*) and *Galaxea* were selected for colony‐scale analyzes in this study. All *Acropora*, *Pocillopora*, and *Galaxea* colonies in each transect at each time point were segmented on the orthomosaics using the positive/negative tool in TagLab (Pavoni et al. 2022) (Figure 1c,d). Due to the generally slower growth rates of massive corals (Buddemeier and Kinzie 1976; Darling et al. 2012; Pratchett et al. 2015), an 80 cm^2^ minimum colony area was applied to *Porites* and *Goniastrea* as a practical threshold for manual segmentation, focusing effort on colonies for which changes in area were more likely to be detectable over the study period while reducing the annotation effort associated with numerous small and fragmented colonies. All taxa were capped at 100 colonies per reef to balance statistical power with time constraints, and colonies were selected at random if there were more than 100 colonies per reef per taxon. Each colony was matched over time and annotated with a bleaching ranking following Gleason (1993). Annotation tables were exported from TagLab and used in downstream analyzes.

Three metrics of structural complexity were extracted for each transect. Fukunaga, Burns, et al. (2020) showed that either fractal dimension or Vector Ruggedness Measure (VRM) at 1, 2, or 4 cm resolution and curvature at 1 cm resolution together effectively capture coral reef structural complexity. Therefore, we used the compound morphometric terrain analysis tool in SAGA GIS v9.8.1 (Conrad et al. 2015) to extract average slope and profile curvature (Zevenbergen and Thorne 1987) and VRM (Sappington et al. 2007) at 1 cm resolution per transect, metrics that have been shown to capture different aspects of the three‐dimensional habitat structure of coral reefs (Fukunaga and Burns 2020; Fukunaga, Burns, et al. 2020; Lazarus and Belmaker 2021; Ferreira et al. 2023). These metrics were extracted from DEMs at *T*_0_ and *T*_3_ to estimate overall change across the study period. Importantly, an average slope and VRM decrease over time indicates a loss of structural complexity. Whereas directional profile curvature, whereby a negative change indicates an increase in convexity or less concavity while a positive change signifies an increase in concavity or less convexity, indicates a change in shape rather than an explicit loss in habitat complexity (Magel et al. 2019; Pascoe et al. 2021; Ferreira et al. 2023). Due to the challenges associated with accurately georeferencing underwater transects, SfM models were not spatially aligned across time points. Transect boundaries were defined within each SfM model using permanent field markers, which were visible in the imagery at each time point. Therefore, the same physically marked area was delineated at each corresponding time point of the SfM models. Furthermore, Raoult et al. (2017) demonstrated low measurement error and no observer or temporal effects, supporting the reliability of SfM models for temporal comparisons. In addition, we also quantified reconstruction repeatability by selecting three transects and rebuilding SfM models and DEMs using the same imagery and methodology. Structural complexity metrics were recalculated for each reconstruction, averaged, and two standard deviations added to obtain an estimate of reprojection error following methodology adapted from Bland and Altman (1986) and Lim et al. (2022).

### 2.5 Data Analyses

All statistical analyzes were conducted in R v4.3.1 (R Core Team 2022) using a three‐phase approach to link thermal stress, biological response, and reef structural change. In the first phase, we modeled how in situ thermal stress related to bleaching response. The colony tracking data exported from TagLab were first filtered to exclude coral colonies at time points with *complete mortality*, when a colony did not have any remaining live tissue, and *bleaching* was defined as when a colony was at least 10% bleached (Gleason 1993). Time points with complete mortality were excluded because bleaching severity could not be assessed after complete loss of living tissue. A generalized additive model (GAM) was then run to model bleaching probability as a function of fixed factors taxa, reef, and a separate smooth function of DHW for each taxon was fitted with model formula *Bleaching ~ s(DHW, by = Taxa) + Taxa + Reef + s(Colony, bs = “re”)* and family binomial. Predicted probabilities and 95% confidence intervals of bleaching by taxa and reefs were then plotted as a function of DHW, and the DHW value at which each taxon and reef had an 80% probability (between likely and very likely) of exhibiting bleaching and 95% confidence intervals were estimated (Virgen‐Urcelay and Donner 2023). To address potential non‐independence among colonies sharing transect‐level thermal exposure, we also fitted an additional model including transect as a random factor; however, this model produced similar outcomes (Table A4), and therefore the more parsimonious model was retained. Pairwise comparisons across reefs and coral taxa were evaluated using the package emmeans v1.10.5 (Lenth and Piaskowski 2025) with DHW fixed at 8°C‐weeks, a threshold defined by NOAA's CRW after which “reef‐wide bleaching and mortality of heat sensitive corals is likely” (NOAA CRW 2025).

In the second phase, we linked environmental heat stress and bleaching response to quantitative change in coral colony area. First, from the colony tracking data exported from TagLab, the total area for each colony was summed at each time point, so that if a colony split, the total area for all fragments was treated as a single unit. The total area of all colonies was then averaged by reef and taxa and plotted over time to visualize temporal change. Change in area for each colony between consecutive time points was calculated and divided by initial area to calculate proportional change, providing a relative measure of colony area change that allows changes to be compared among colonies of different initial sizes (Hughes and Jackson 1985; Hughes and Connell 1987; Kayal et al. 2015, 2023; Dornelas et al. 2017). Time periods between each consecutive time point were defined in relation to the time of occurrence of the 2023 bleaching event in the study area as Pre1 (pre‐bleaching, *T*_0_ to *T*_1_), Pre2 (pre‐bleaching, *T*_1_ to *T*_2_), and Post (post‐bleaching, *T*_2_ to *T*_3_). Time points with complete mortality were retained for this analysis, where complete mortality was represented by complete loss of living colony area. Another GAM was then used to model proportional change of colony area as a function of fixed factors taxa, lagged DHWs, lagged bleaching ranking, and taxa and time period interaction, with reef and colony as random factors to account for repeated measures. DHW and bleaching ranking were lagged, meaning that the value for the previous survey time point was used in the model. The model formula was Proportional change ~ Taxa * Timeperiod + s(Lagged DHW, by = Taxa, k = 9) + s(Lagged Gleason, by = Taxa) + s(Reef, bs = “re”) + s(Colony, bs = “re”) with family gaussian. Predicted proportional changes in colony area were plotted and pairwise comparisons were estimated using the package emmeans v1.10.5 (Lenth and Piaskowski 2025).

For the third phase linking coral colony‐scale area change with reef‐scale structural complexity, the overall change in area for each taxon was estimated by subtracting the area at *T*_0_ from the area at *T*_3_. *Galaxea* was excluded from this analysis due to the relatively small sample size across reefs. The absolute change in colony area was used in this phase rather than proportional change as in phase two to reflect the overall physical contribution of colonies to reef structure. Similarly, the overall change in reef structural complexity metrics for each transect at the start and end of the study period was calculated. Overall, changes in reef structural complexity from the start to the end of the study period were used in this analysis as we expect that changes in reef‐scale structure to occur as cascading, longer‐term effects (Sheppard et al. 2002; Graham et al. 2007, 2015; Pratchett et al. 2008; Alvarez‐Filip 2010; Bozec et al. 2015; Roff et al. 2020). Variables were standardized using z‐scores and a correlation matrix was inspected to assess potential redundancy among variables prior to their inclusion in a principal component analysis (PCA), with no pairwise correlations exceeding *r* = 0.8 (Guillaume et al. 2026). A PCA was then performed to characterize covariation among reef structural variables and colony area per taxon.

## 3 Results

### 3.1 Orthomosaic Properties and Coral Communities

A total of 71 orthomosaics at 1 mm resolution and 35 DEMs at 1 cm resolution, each covering a 100 m^2^ area, were generated from 106,343 digital images for the 18 transects surveyed from *T*_0_ to *T*_3_ (excluding one transect at *T*_0_ at Tahla). The median root mean square error (RMSE) of the models was 0.0016 m (interquartile range: 0.0008 m–0.0047 m). Across the 6 reefs at *T*_0_, benthic cover was estimated using the point‐intercept method applied to orthomosaic imagery and revealed that hard coral cover averaged 21% and ranged from less than 10% on Al Fahal (midshelf) and Shib Nazar (offshore) to nearly 40% on Monument (inshore) and Rose Reef (offshore) (Figure A1). Massive coral colonies were generally more dominant across reefs than branching ones, particularly on Monument (inshore) and Shib Nazar (offshore), with the exception of Al Fahal (midshelf), where the branching coral taxa were more abundant than the massive ones. Collectively, *Pocillopora*, *Acropora*, *Porites*, and *Goniastrea* comprised about 57% to 100% of the scleractinian coral cover across reefs and were therefore targeted for colony‐scale analyzes in addition to *Galaxea*. A total of 1036 unique colonies of these five target taxa at *T*_0_ were segmented and fate‐tracked across the study period. Figures A2 and A3 show the total number of colonies tracked at each time point and the size frequency distributions over time.

### 3.2 Probability of Bleaching Across Target Taxa as a Function of DHW

In situ temperature metrics obtained from the data loggers revealed spatial and temporal variation in thermal exposure across reefs during the study period. Specifically, average DM and DHW extracted from in situ loggers across reefs between September 2022 and April 2024 ranged from 25.0°C to 34.4°C and 6.2°C‐weeks to 23.5°C‐weeks, respectively (Figure 2). Temperatures and DHW peaked in summer 2023, coinciding with the bleaching event documented in this study and remained elevated through December 2023. Across reefs, DHW values spanned a broad range over time, indicating substantial variation in cumulative thermal stress experienced during the study period.

**FIGURE 2:**
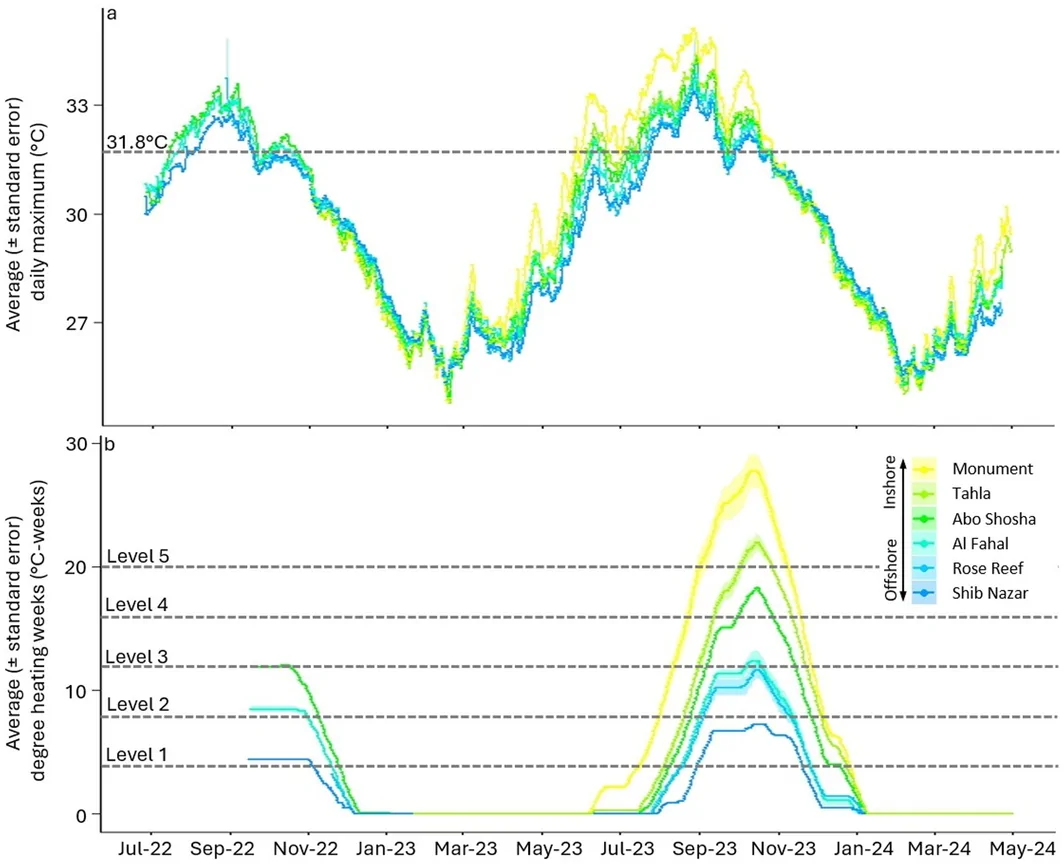
In situ heat stress across the 6 study sites in the central Saudi Arabian Red Sea collected with Star Oddi DST CT loggers from September 2022 to April 2024. (a) Average (± standard error) daily maximum (°C) with the dotted line showing the putative coral bleaching threshold for the region (31.8°C; NOAA CRW 2019) and (b) average (± standard error) degree heating weeks (DHW; °C‐weeks) per reef from inshore to offshore with NOAA bleaching alert levels shown by the dotted lines (NOAA CRW 2025).

Bleaching probability significantly differed among coral taxa and reefs, with differences in both the magnitude and shape of responses to increasing DHW. The GAM (*n* = 2662) modeling the probability of bleaching across reefs and taxa as a function of in situ DHW had an *R* ^2^ value of 0.587 (Figure 3), which included repeated measures of the tracked colonies over time. The DHW threshold for 80% probability of bleaching for taxa ranged from *Galaxea* at 7.2°C‐weeks (95% CI: 5.6°C–8.9°C‐weeks) to *Goniastrea* at 10.8°C‐weeks (95% CI: 9.4°C–11.9°C‐weeks), and reefs ranged from 8.9°C‐weeks (95% CI: 7.5°C–10.3°C‐weeks) at Rose Reef (offshore) to 15.0°C‐weeks (95% CI: 13.3°C–16.9°C‐weeks) at Abo Shosha (inshore) (Table 1). When DHW were fixed at 8°C‐weeks (NOAA CRW 2025), the probability of bleaching significantly differed between coral genera groupings: (1) *Galaxea* and *Porites* with higher probabilities of bleaching and (2) *Pocillopora* and *Goniastrea* with lower probabilities, with *Acropora* exhibiting intermediate levels and not significantly from either group (Figure 3a; *p*‐values for pairwise comparisons are reported in Table A1). Furthermore, the shape of the curve of *Pocillopora* and *Goniastrea* exhibited a threshold response, with a relatively flat slope at low DHW, followed by a steep increase before plateauing. In contrast, *Porites* and *Galaxea* showed a positive but less steep slope starting from low DHW, while *Acropora* showed an intermediate relationship. The probability of bleaching significantly differed between reef groupings: (1) Tahla, Rose Reef, and Shib Nazar with higher probabilities of bleaching at 8°C‐weeks and (2) Al Fahal and Abo Shosha with lower probabilities, with Monument at intermediate levels and not significantly different from either group (Figure 3b; *p*‐values for pairwise comparisons are reported in Table A1).

**FIGURE 3:**
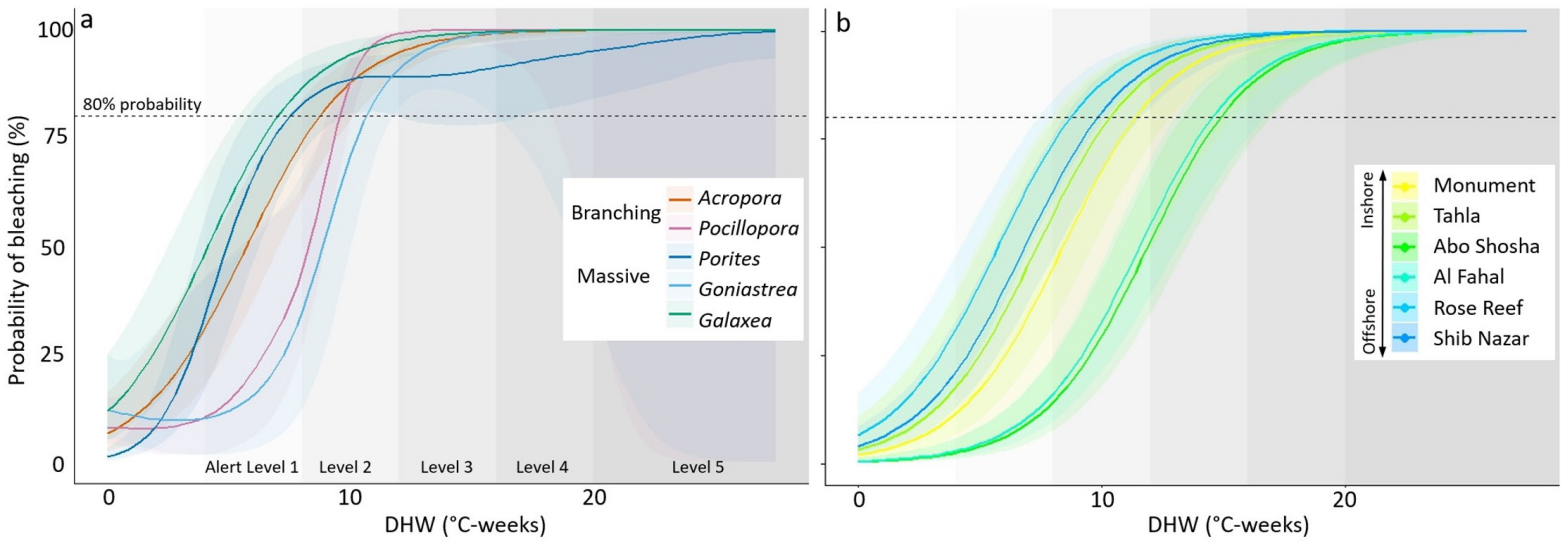
Predicted probability of coral bleaching with 95% confidence intervals (shaded areas) by (a) scleractinian taxon and (b) reef as a function of degree heating weeks (DHW; °C‐weeks) across the 6 study sites in the central Saudi Arabian Red Sea in September 2023. Gray shaded areas indicate National Oceanic and Atmospheric Administration's Coral Reef Watch Bleaching Alert Levels (NOAA CRW 2025).

**TABLE 1:** Estimated degree heating week (DHW; °C‐weeks) thresholds for 80% probability of bleaching and associated 95% confidence intervals (CI) by scleractinian coral taxa and reef across the 6 study sites in the central Saudi Arabian Red Sea from September 2022 to April 2024 (extracted from the model in Figure 3).

	DHW80	95% CI
Taxa		
*Galaxea* 1	7.2	5.6–8.9
*Porites* 1	7.5	5.8–9.7
*Acropora* 1,2	8.9	7.5–10.3
*Pocillopora* 2	9.7	9.2–10.3
*Goniastrea* 1,2	10.8	9.4–11.9
Reef		
RoseReef 1	8.9	7.5–10.3
ShibNazar 1	10.0	8.6–11.7
Tahla 1	10.5	8.3–12.8
Monument 1,2	11.7	7.8–15.3
AlFahal 2	14.7	13.0–16.9
AboShosha 2	15.0	13.3–16.9

*Note:* Numbers next to names indicate significantly different groupings with DHW fixed at 8°C‐weeks.

### 3.3 Change in Colony Area Across Target Taxa

During the study period, there was an estimated net loss of 13.25 m^2^ of live coral area in total across the 5 taxa and 6 study reefs. The percent loss of live colony area across all reefs was particularly severe for branching taxa *Acropora* and *Pocillopora* (78% and 74% on average, respectively) and *Galaxea* (71%). Massive *Porites* exhibited smaller losses in general (33% on average), and massive/columnar *Goniastrea* showed intermediary levels (63% on average), though both taxa still showed substantial reductions on more inshore reefs (up to 98%) (Figure 4). *Pocillopora* and *Goniastrea* were the only two taxa that exhibited net increases on some reefs like Al Fahal (midshelf) and Shib Nazar (offshore). In general, declines in colony area across all five taxa decreased with increasing distance from shore.

**FIGURE 4:**
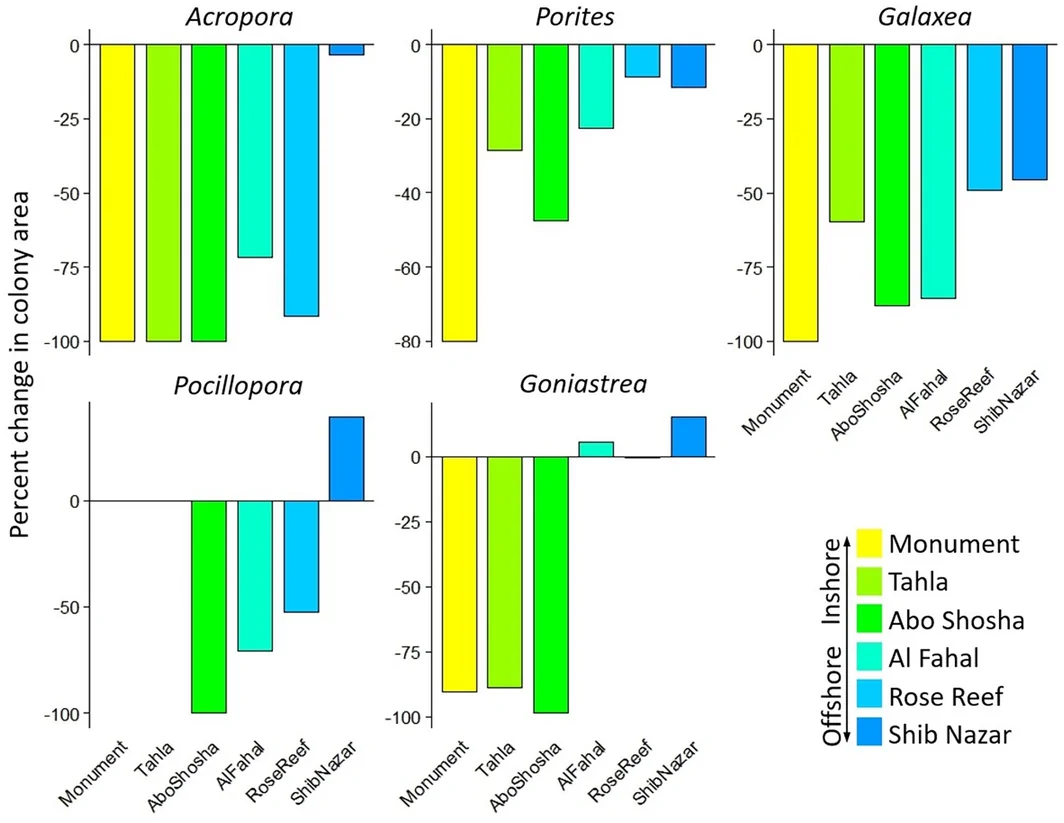
Net percent change in coral colony area (%) across the five scleractinian taxa and study reefs in the central Red Sea from September 2022 to April 2024.

At a finer temporal scale between surveys, in Pre1 (before the 2023 bleaching event), *Acropora*, *Pocillopora*, *Porites*, and *Galaxea* all exhibited net growth across all reefs, while *Goniastrea* exhibited net loss in area (Figure A4). However, in Pre2 and Post, all taxa exhibited a net loss in area. The greatest absolute growth between two consecutive time points across reefs and taxa was *Porites* on Rose Reef (+3311 cm^2^) and Tahla (+2107 cm^2^) in Pre1, followed by *Pocillopora* on Al Fahal in Pre1 (+2062 cm^2^). The greatest absolute loss between two consecutive time points across reefs and taxa was *Pocillopora* on Al Fahal in Post, following the bleaching event (−9150 cm^2^; i.e., Figure 1d), followed by *Porites* in Pre2 on Abo Shosa (−6512 cm^2^), and *Porites* in Post on Rose Reef (−3691 cm^2^).

The relationship between changes in colony area following the 2023 bleaching event and in situ DHW and bleaching severity were taxon‐specific. The GAM (*n* = 2057) that modeled proportional change in colony area had an *R* ^2^ value of 0.273 (Figure 5). *Acropora, Pocillopora, Galaxea*, and *Porites* showed significantly higher change in area (shrinkage) in Post (after the 2023 bleaching event) compared to Pre1 (*p* < 0.05, reported in Table A2). DHW strongly explained changes in colony area for *Acropora*, *Pocillopora*, and *Porites* (Figure 5a; *p* < 0.05, reported in Table A2), and exhibited steep negative slopes for the branching taxa, while *Porites* had a non‐linear relationship (Figure 5a; partial effects plots are shown in Figure A5). Bleaching ranking explained additional variation in change in colony area of *Pocillopora* after accounting for DHW (Figure 5b; *p* < 0.001, reported in Table A2). DHW and bleaching ranking were not significant explanatory variables for change in colony area of *Goniastrea* and *Galaxea* (*p* > 0.05), and are therefore not displayed in Figure 5. Reef was a significant term in the model (*p* < 0.001), while colony was not significant (*p* = 0.419).

**FIGURE 5:**
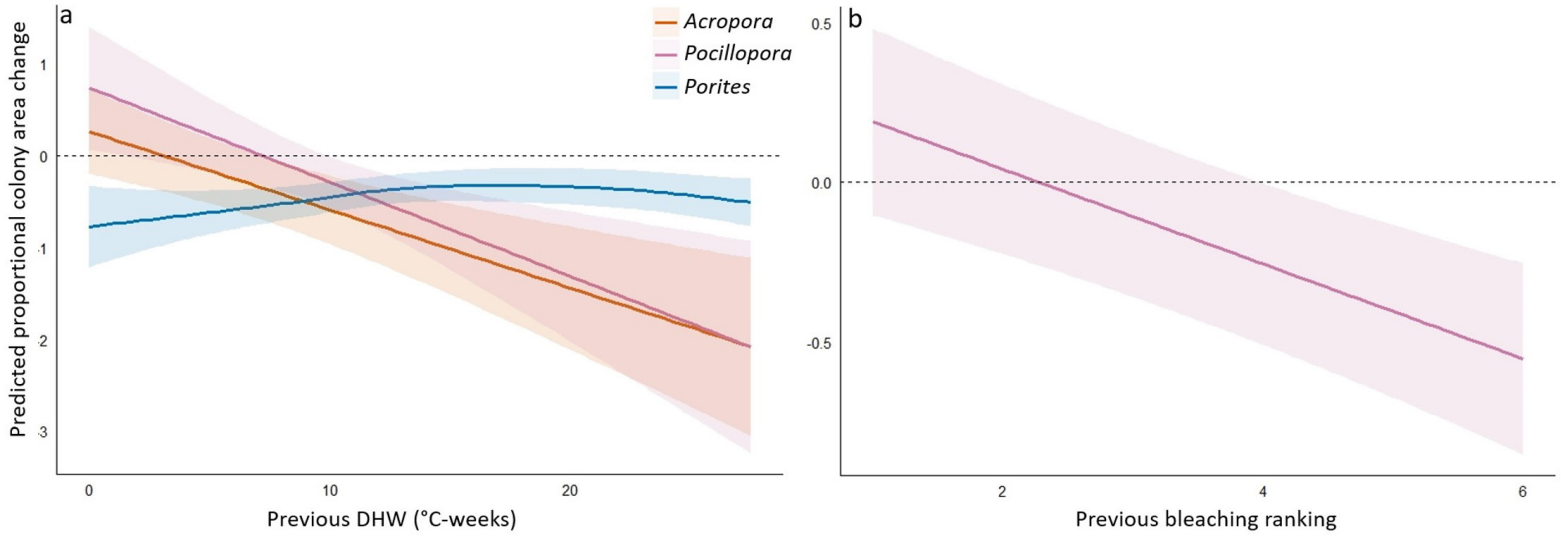
Predicted proportional change in total coral colony area for each taxon across the 6 study sites in the central Saudi Arabian Red Sea from September 2022 to April 2024 modeled as a function of lagged (a) degree heating weeks (DHW; °C‐weeks) and (b) bleaching ranking (Gleason 1993) after accounting for DHW. Only taxa in which DHW and bleaching ranking significantly predicted change in colony area are shown. The dotted lines indicate no change.

### 3.4 Change in Reef Structural Complexity

Most estimates of changes in coral reef structural complexity metrics across transects were greater than the error envelope calculated (Figure 6), indicating that estimated changes were likely real‐world. Across reefs, structural change over the study period was characterized by a general reduction in slope, indicating overall flattening of vertical relief (Figure 6). Changes in VRM were more variable, with most reefs showing slight declines, indicating a general smoothing of fine‐scale structural complexity, although some transects exhibited increases (Figure 6; e.g., Rose Reef). Profile curvature showed a wide range of variability, with some reefs increasing in concavity while others increased in convexity. Specifically, Abo Shosha (inshore) and Al Fahal (midshelf) showed overall slope reductions across transects and declines in VRM, indicating consistent flattening and general smoothing. Abo Shosha exhibited increased convexity, whereas Al Fahal showed mixed curvature responses. Monument (inshore) and Tahla (inshore) displayed contrasting changes in slope and VRM across transects, with Tahla increasing in concavity and Monument showing mixed curvature responses. Rose Reef (offshore) exhibited relatively small and mixed changes in slope, with VRM increases at most transects and an increase in concavity overall. Shib Nazar (offshore) also experienced small and variable slope changes across transects, but VRM decreased overall, with most transects increasing in convexity.

**FIGURE 6:**
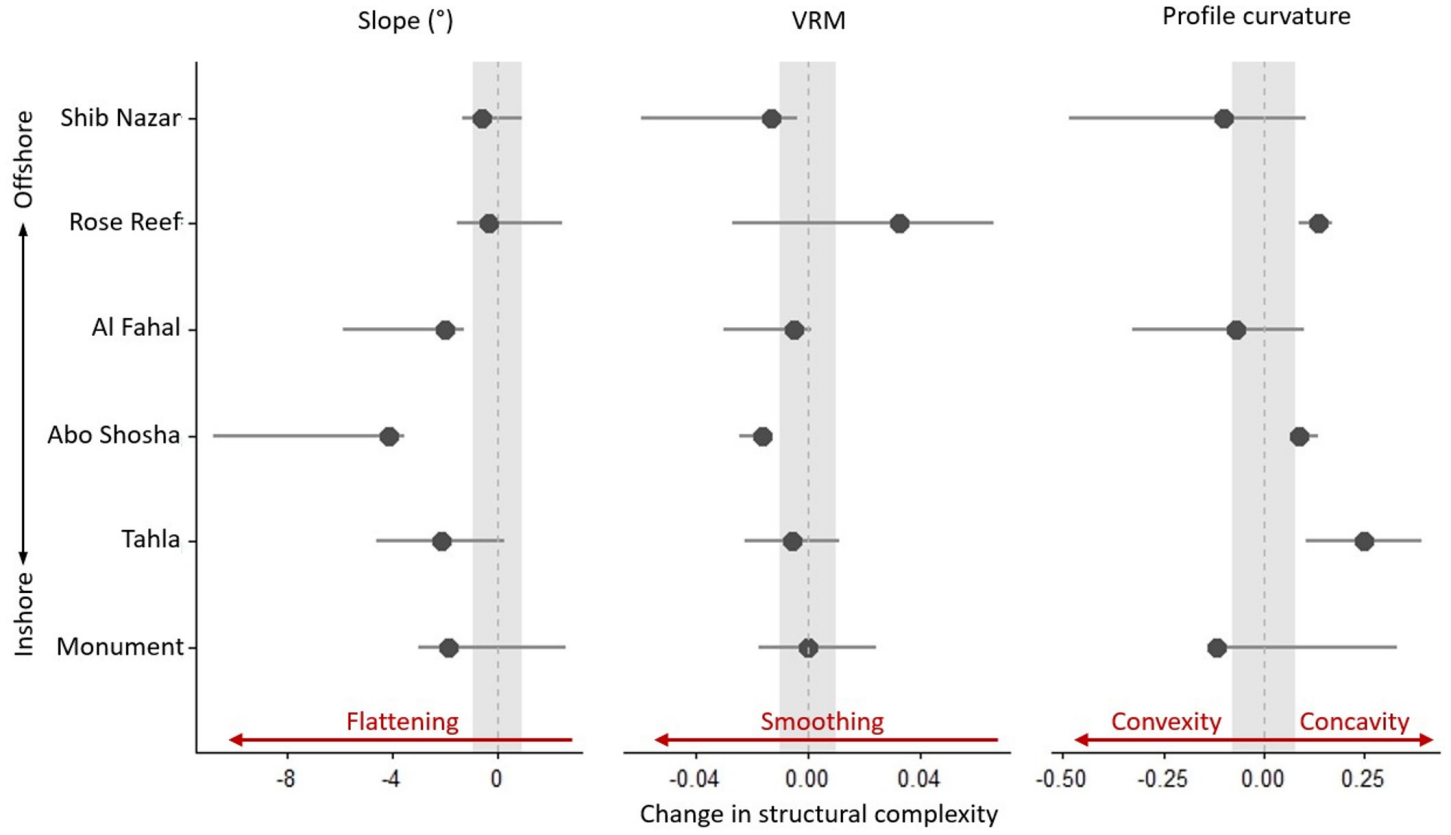
Overall median, minimum, and maximum change in different metrics of structural complexity across the 6 study reefs in the central Red Sea from September 2022 to April 2024. Gray shaded area indicates the reprojection error that was estimated by recalculating the metrics on three of the re‐generated digital elevation models, averaged plus and minus two standard deviations.

The PCA revealed that changes in structural complexity were multidimensional and associated with branching and massive coral morphologies (Figure 7). We found that the first two axes of the PCA explained 58.2% of the total variation in the dataset. The first principal component axis (PC1) was associated with a loss in massive *Porites* and *Goniastrea* and decreased slope, explaining 34.8% of the variation (loadings were 0.386, 0.586, and 0.554 respectively, and are shown in Table A3). The second principal component axis (PC2) was associated with a loss in branching corals *Acropora* and *Pocillopora* and profile curvature, explaining 23.4% of the variation (loadings were 0.605, 0.550, and 0.452 respectively, and are shown in Table A3).

## 4 Discussion

This study used a multi‐phase modeling approach to quantify the link between the 2023 thermal stress, scleractinian coral bleaching and mortality, and coral reef structural complexity in the central Red Sea. Reef‐scale variability in bleaching sensitivity suggested that the ecological risk associated with regional alert thresholds can vary substantially among reefs in the central Red Sea. We further support that the relationship between thermal stress and change in colony area is taxon‐dependent, consistent with previous studies (Dias et al. 2018; Pisapia et al. 2019; Speare et al. 2022; Khen et al. 2024; Álvarez‐Noriega et al. 2025). This study also represents the first quantitative, taxon‐specific assessment of how bleaching‐driven mortality translates to reef‐scale structural degradation in the central Red Sea, and presents a scalable framework that enables fate tracking of individual colonies to model bleaching, mortality, and changes in structural complexity.

### 4.1 Taxon‐ and Reef‐Specific Bleaching Thresholds in the Central Saudi Arabian Red Sea

The GAM modeling the probability of bleaching explained a substantial proportion of the variation in the dataset, suggesting that in situ DHW was a strong explanatory variable of bleaching in general. However, the remaining unexplained variance suggests that additional biological and/or environmental factors may also influence bleaching responses. For example, one limitation of this model is that although in situ DHW were used, these were based on the daily minimum values to align with NOAA methodology (NOAA CRW 2018), whereas actual heat exposure was likely much higher as demonstrated by DM (Figure 2a). In the historical context, temperatures were higher in 2023 compared to previous bleaching events (Bähr et al. 2025; Figure S3); however, temperature stress was higher the following year (2024) (Dunn et al. 2026; Figure 2), whereby coral responses in 2023 were possibly stronger relative to historical events but less severe than those observed in 2024.

The probability of bleaching increased with DHW across all five taxa, however responses varied from more rapid increases in bleaching probability to more gradual increases, with no consistent pattern distinguishing branching from massive taxa. While 95% confidence intervals were relatively large in some cases, significant differences were still observed among taxa. This suggests that among these Red Sea coral genera, growth form is insufficient to explain bleaching vulnerability, in contrast with previous studies (Loya et al. 2001; McClanahan 2004; van Woesik et al. 2011; Manzello et al. 2025). These differences reflect variation in how thermal stress is accumulated and expressed physiologically across taxa, namely how heat stress leads to cumulative damage to symbiont photosynthetic systems and the buildup of oxidative stress, ultimately triggering bleaching (Lesser 1997; Weis 2008). These differences may also be in part related to differences in symbiont community composition (Baker et al. 2004; LaJeunesse et al. 2010). Furthermore, differences in thermal history of local environmental conditions can also mediate bleaching responses (McCarthy et al. 2024). Microhabitat variability may further modulate realized thermal exposure, creating spatial heterogeneity in heat stress even within similar DHW levels (Lenihan et al. 2008). However, at the population‐level across study reefs, all five taxa had relatively high bleaching probabilities within bleaching alert Level 1 or 2, aligning with NOAA CRW regional warnings.

DHW thresholds varied across reefs. At the reef‐scale, the lowest DHW threshold with 80% probability of bleaching was Rose Reef (offshore; 8.9°C‐weeks), which corresponds to NOAA bleaching alert Level 2 (8°C‐weeks to 12°C‐weeks) expecting “reef‐wide bleaching with mortality of heat sensitive corals”. Offshore reefs in general reached high bleaching probabilities at lower DHW; however, some more inshore reefs experienced higher accumulated heat stress without reaching similarly high bleaching probabilities, potentially reflecting acclimatization to their historically warmer thermal regimes. However, acclimatization was not directly measured, and variation in bleaching susceptibility among reefs could also be explained by relative community susceptibility, a measure of the bleaching susceptibility of each taxon and its relative abundance, depth, local hydrodynamics, reef morphology, substrate, or logger position (Couch et al. 2017). Indeed, as taxon abundances differed among reefs (Figure A1), the reef effect and community‐composition effect are difficult to separate. For example, the coral community on Al Fahal was dominated by *Pocillopora*, a genus represented by one endemic species in the Red Sea (Oury et al. 2025), at *T*_0_ (Figures A1 and A2), which, contrary to previous findings (Loya et al. 2001; McClanahan 2004; van Woesik et al. 2011; Manzello et al. 2025), exhibited one of the highest DHW80 thresholds for bleaching and could in part explain the higher DHW threshold of Al Fahal. Overall, reef‐level DHW thresholds varied by more than 6°C‐weeks and most were higher than Rose Reef (> 10°C‐weeks), indicating substantial spatial heterogeneity in bleaching sensitivity that is not captured by uniform satellite‐based alert thresholds. This indicates that bleaching alert levels may represent markedly different levels of ecological risk depending on reef‐specific sensitivity, with some reefs already experiencing high bleaching probabilities at or below the alert window, while others remain comparatively resistant. However, rather than establishing a single revised bleaching threshold, we emphasize that our results instead support refining remote‐sensing products at the reef‐scale to complement existing NOAA CRW products by empirically linking regional thermal stress metrics to in situ biological responses in the central Red Sea. It is also important to highlight here that a high propensity to bleach does not necessarily translate to high mortality (McClanahan 2004; Baker et al. 2008; Berkelmans 2009; Dunn et al. 2025).

### 4.2 From Bleaching to Loss in Living Coral Area Across Target Taxa

The GAM modeling proportional change in colony area explained nearly 30% of the variation in the dataset. Although proportional change standardized area loss or gain relative to initial colony size, size‐dependent processes may still contribute to variation in subsequent growth or area loss that was not explicitly modeled here. Nevertheless, *Acropora* displayed the clearest coupling between thermal stress and reduction in living coral surface, whereby in situ DHW was a strong predictor of change in colony area. Bleaching severity provided little extra information, suggesting it may be tightly coupled with DHW for this taxon. DHW was also a strong predictor of change in living colony surface for *Pocillopora*, however bleaching severity explained additional variation in the dataset, suggesting that DHW is necessary but not sufficient to explain change in living colony surface. This suggests that bleaching severity and subsequent change in colony area can vary among *Pocillopora* colonies at similar DHWs. DHW was a significant predictor of change in colony area for *Porites* as well, however the relationship was not linear and reduction in living coral surface mainly occurred at higher levels of cumulative heat stress. Therefore, for this taxon, DHW has explanatory value but the relationship is complex and limited, whereby an increase in DHW may not lead to reduction in living coral surface. DHW was not a significant explanatory variable of change in colony area for *Goniastrea* or *Galaxea*. For the latter, this may reflect limited statistical power due to a small sample size. However, the lack of a DHW effect for *Goniastrea* suggests that change in colony area was decoupled from thermal stress over the observed DHW range, such that DHW may have induced stress without translating into measurable area loss on this timescale. Notably, *Goniastrea* was the only taxon to exhibit an overall reduction in living colony area during Pre1, illustrating that net coral area loss occurred prior to the bleaching event and was not offset by growth, unlike for fast‐growing branching taxa. This pre‐existing decline may have obscured any relationship between DHW and subsequent changes in colony area. The relationship was not obscured with *Porites*, although also a slow‐growing massive coral, possibly because this genus can exhibit higher growth rates than *Goniastrea* (Highsmith 1979) and a strong capacity for micro‐colony fusion (Forsman et al. 2015), consistent with our observations of tissue infilling in larger colonies (e.g., Figure A6). Together, these taxon‐specific responses are consistent with differences between bleaching resistance and short‐term tissue‐area persistence, and demonstrate different strategies potentially relating to thickness of tissue (Loya et al. 2001), metabolism (Gates and Edmunds 1999), energy reserves (Rodrigues and Grottoli 2007), or symbiont flexibility (Ziegler et al. 2015; Terraneo et al. 2024).

At the reef‐scale, offshore reefs experienced lower heat stress than inshore reefs in 2023, consistent with previous bleaching events in the region (Furby et al. 2013; Monroe et al. 2018), and subsequently showed smaller net losses in colony area, with some sites even exhibiting gains. This suggests that offshore reefs may have provided shelter during this thermal stress event, as observed in previous bleaching events (Furby et al. 2013; Monroe et al. 2018). However, during the successive 2024 bleaching event, offshore reefs were impacted as severely as inshore reefs, suggesting that such refuge may no longer persist under increasing climate change (Dunn et al. 2026).

One strength of this dataset is that the two‐dimensional surface area of each colony was quantified, enabling a more precise estimation of colony size compared to maximum dimension (Ferreira et al. 2023). However, as methodological improvements with machine learning and artificial intelligence streamline colony annotations, further research could investigate the complete three‐dimensional structure of each colony. One limitation of our model was that while changes in total coral colony area were captured by summing area across fragments in the case of colony splitting, this approach did not distinguish between reductions in size due to shrinkage versus fission, which may have different ecological implications and could add another layer of analysis for further investigation (Hughes and Jackson 1980). It is also important to emphasize that the overall loss in living area across the five taxa reaching 13.25 m^2^ during the study period was focused solely on the original tagged colonies, and the transects were not surveyed for new recruits; therefore, the overall net loss of coral per reef may be an overestimation.

### 4.3 Reduction in Living Coral Surface Following Bleaching Relates to Measurable Changes in Reef Structural Complexity

Our results show that structural change following bleaching is detectable but nuanced in a relatively short timeframe. Estimation of reconstruction error via repeated SfM models provided a conservative uncertainty envelope of structural complexity metrics, with most observed values exceeding this range, indicating that detected changes were unlikely to be artifacts of SfM reconstructions. However, these estimates did not capture potential errors arising from differences in image acquisition, camera trajectory, visibility, or GCP placement, which may also influence SfM‐derived structural complexity measurements (Bryson et al. 2017). Across reefs, structural change generally reflected flattening of vertical relief, smoothing of fine‐scale complexity, and shifts in concavity/convexity. However, in contrast to catastrophic disturbances such as storms, which often result in clear and uniform reduction in structural complexity (Burns et al. 2016; Pascoe et al. 2021), the changes detected here were in some cases subtle or contradictory among transects within the same reef. Changes in individual metrics may also reflect multiple ecological processes and can be challenging to interpret. For example, while most reefs exhibited a decrease in VRM, Rose Reef showed increases across some transects potentially reflecting an increase in rubble, rather than an increase in biogenic complexity (Pascoe et al. 2021; Figure A7). In addition, as second derivatives, curvature metrics are particularly sensitive to reconstruction noise, and positive changes in profile curvature may indicate either increased concavity (e.g., hole formation) or reduced convexity (e.g., erosion of protruding structures), and conversely negative changes may reflect reduced concavity (e.g., holes filling in) or increased convexity (e.g., loss of interstitial spaces due to branch breakage), corresponding to opposing ecological interpretations (Pascoe et al. 2021; Ferreira et al. 2023). Nevertheless, the changes detected in this study occurred 7 months after the bleaching event and during less than 2 years in total, which is rapid relative to multi‐year or decadal timescales of structural change (Sheppard et al. 2002; Pratchett et al. 2008; Alvarez‐Filip 2010; Bozec et al. 2015; Graham et al. 2015; Roff et al. 2020) and local net‐erosion/accretion rates (Roik et al. 2018), but is in line with other recent studies using a similar methodology (Couch et al. 2017; Kopecky et al. 2023). It is important to highlight that this timeframe may not capture slower processes of erosion. Coral mortality may not result in rapid loss of physical structure, such that declines in colony area are not necessarily accompanied by detectable changes in structural complexity.

Changes in reef structure following bleaching were captured by multiple complementary metrics, which revealed distinct associations between coral growth forms and components of structural complexity. While Alvarez‐Filip (2010) found that structural complexity did not significantly change following bleaching, the study characterized structural complexity by a single rugosity index, which may not accurately capture the different dimensions of reef structural complexity. Indeed, our PCA results indicated that curvature, representing fine‐scale geometric properties, captured patterns of variation distinct from slope. Across reefs, a loss of overall living coral surface of branching growth forms (*Acropora, Pocillopora*) was consistently associated with changes in profile curvature, reflecting alterations to fine‐scale microhabitat and interstitial spaces. This pattern was evident at Al Fahal, where susceptible branching corals were dominant over massive morphologies at *T*_0_ (Figures 1d, 7, and A1), and increased convexity likely reflects a loss of interstitial spaces due to branch breakage (Ferreira et al. 2023). In contrast, massive colonies were associated with changes in slope, indicating alterations to broader‐scale reef structure, as observed at Abo Shosha (Figure 7). Massive corals grow and erode slowly and generate coarse topography rather than fine‐scale structure (Chappell 1980; Jackson and Hughes 1985; Edinger and Risk 2000; Fukunaga, Burns, et al. 2020; Helder et al. 2022; Ferreira et al. 2023). While they contribute to long‐term stability, they do not compensate for the fine fractal complexity lost following bleaching‐induced mortality of branching taxa. Together, these results support the multidimensional nature of structural change, and while specific coral morphologies have been associated with distinct components of reef structural complexity (Burns et al. 2015, 2016; Magel et al. 2019; Fukunaga and Burns 2020; Fukunaga, Burns, et al. 2020; Pascoe et al. 2021; Ferreira et al. 2023) and bleaching has led to a loss of structural complexity (Graham et al. 2007; Couch et al. 2017; Kopecky et al. 2023), this study further supports that structural degradation following bleaching is taxon‐specific. However, metric‐taxon relationships can vary among regions (Burns et al. 2019; Fukunaga, Burns, et al. 2020; Ferreira et al. 2023; Mills et al. 2023), precluding generalization across systems. Instead, our findings reinforce that multiple metrics are required to effectively characterize the reef habitat. It is also important to note that while the PCA demonstrated multivariate covariation, it does not directly test whether coral area loss predicts changes in structural complexity.

**FIGURE 7:**
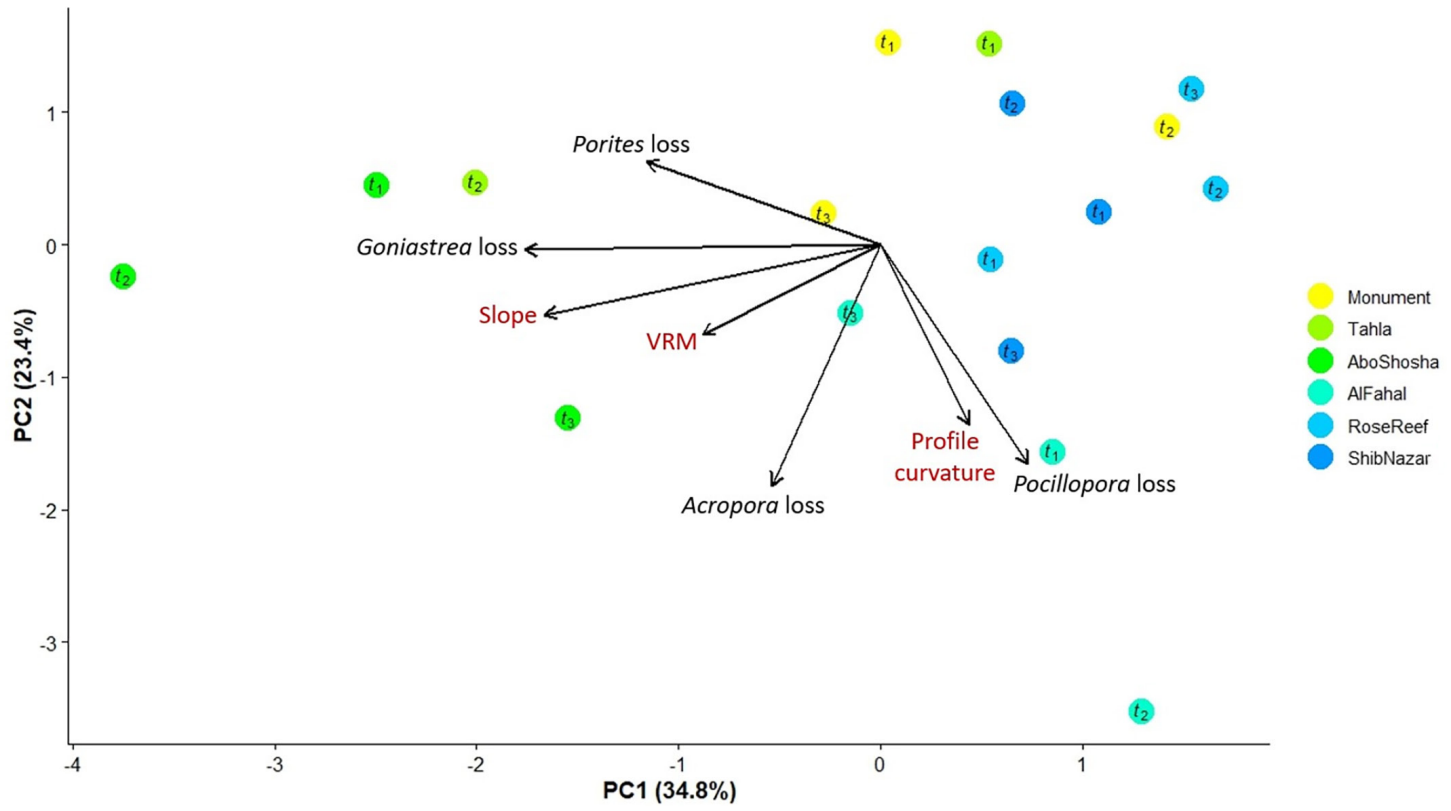
Principal component analysis of overall change (from the first time point in September 2022 to the last in April 2024) in total coral colony area of each taxon and structural complexity for each replicate transect (*t*_1–3_) across the 6 study sites in the central Saudi Arabian Red Sea. The direction of vectors indicates increase loss (negative change).

Beyond taxonomic identity and overall loss of coral (Figure A2), structural change may also be influenced by shifts in size frequency distributions. Post‐bleaching communities were dominated by smaller colonies (Figure A3), and such shifts have been shown to reduce structural complexity (Pereira and Munday 2016; Richardson, Graham, and Hoey 2017; Urbina‐Barreto et al. 2021; Ferreira et al. 2023). More broadly, structural change has been shown to be scale‐ and resolution‐dependent, with relationships varying with plots size, survey methodology, and geographic location (Wedding et al. 2011; Fukunaga and Burns 2020; Fukunaga, Burns, et al. 2020; Ferreira et al. 2023). In addition, the substratum can also influence structural complexity (Nash et al. 2013; Fukunaga, Burns, et al. 2020; Asbury et al. 2023; Ferreira et al. 2023). While Abo Shosha (inshore) and Al Fahal (midshelf) exhibited the clearest structural change associated with coral identity, Shib Nazar (offshore), Rose Reef (offshore), and Monument (inshore) clustered on the opposite side of the PCA. This could be in part relating to Abo Shosha and Al Fahal being dominated by low‐relief unconsolidated substrate, including rubble and sandy seabed with discrete patch reefs, while in contrast, Shib Nazar, Rose Reef, and Monument all have consolidated carbonate framework with higher three‐dimensional geomorphological complexity, including features such as pinnacles, vertical walls, and crevice formations (Figure A1). In the first group, structural complexity is likely largely driven by living coral communities, whereas in the former group, structural complexity may be driven by the substratum (consolidated carbonate framework), reducing the detectability of live coral‐driven structural change and potentially conferring greater short‐term structural persistence (Alvarez‐Filip 2010; Ferreira et al. 2023). However, this topic warrants further investigation.

### 4.4 Implications for Reef Structural Persistence in the Central Red Sea

In the context of accelerating climate change, our findings suggest that repeated heatwaves are likely to restructure coral communities through potentially predictable, taxon‐specific mortality (Loya et al. 2001; McClanahan 2004; van Woesik et al. 2011; Manzello et al. 2025) that systematically alter three‐dimensional structural complexity. Within the central Red Sea, reefs dominated by branching corals are expected to lose fine‐scale structural features rapidly and repeatedly under recurrent bleaching. In contrast, reefs dominated by massive corals may retain overall structure for longer, but an eventual loss is likely to result in slower and more persistent reductions in large‐scale reef architecture. While these patterns should further be investigated in the northern and southern regions given their unique conditions (Sofianos and Johns 2015; Ellis et al. 2017; Berumen et al. 2019), this suggests that central Red Sea reefs experiencing similar losses in coral cover can exhibit markedly different structural outcomes depending on which coral taxa are lost, with implications for ecosystem functions. Fine‐scale structural features, reflected by metrics such as profile curvature, are closely linked to juvenile fish refuge, invertebrate habitat availability, and boundary‐layer flow dynamics (Reidenbach et al. 2006; Pratchett et al. 2008; Stella et al. 2011; Graham and Nash 2013; Fukunaga, Kosaki, et al. 2020), whereas broader‐scale structural attributes such as slope influence wave energy dissipation and habitat availability for larger‐bodied organisms (Sheppard et al. 2005; Fukunaga, Kosaki, et al. 2020; Helder et al. 2022). Indeed, a decline in structural complexity in the Caribbean and Indo‐Pacific may be linked to reduced fish biodiversity (Pratchett et al. 2008; Alvarez‐Filip 2010; Newman et al. 2015). Overall, our findings indicate that predicting and monitoring reef structural trajectories must go beyond coral cover and a single metric of structural complexity and requires integrating thermal exposure, coral community composition, and structural metrics across spatial scales.

## 5 Conclusions

This study quantified the link between the 2023 thermal stress, coral bleaching and mortality, and structural complexity at the reef‐scale in the central Red Sea, providing a mechanistic framework for understanding how heat stress translates into changes in reef structure. While population‐level taxon‐specific in situ bleaching thresholds broadly aligned with regional alert levels, ecological risk varied substantially across reefs, indicating that regional thermal metrics alone are insufficient to resolve fine‐scale spatial variability. We then showed that the relationship between thermal stress and change in colony area is taxon‐dependent, supporting that the consequences of bleaching and mortality on structural complexity vary among coral communities. Furthermore, we provide uncertainty‐quantified evidence that bleaching‐driven coral mortality can rapidly alter reef structure in the central Red Sea, reinforcing the direct link between colony‐scale stress responses and habitat degradation. Last, this study represents the first colony‐scale, mechanistic assessment of coral resilience across taxa during an extreme heat stress event in the central Red Sea. Together, these findings show that reef‐scale structural vulnerability to heat stress is governed by taxon‐specific responses that are not captured by regional thermal metrics alone. This highlights a critical limitation in current reef assessments and underscores the need to integrate colony‐scale response data and multidimensional metrics of reef structural complexity. By providing a scalable framework, this study enables future research to integrate colony level bleaching and mortality with reef‐scale structural assessments across systems and environmental gradients. Such approaches will be essential for improving predictions of reef structural persistence under accelerating climate change.

## Author Contributions

**Natalie Dunn:** conceptualization (equal), data curation (equal), formal analysis (equal), investigation (equal), project administration (equal), visualization (equal), writing – original draft (equal), writing – review and editing (equal). **Fabio Marchese:** conceptualization (equal), data curation (equal), resources (equal), supervision (equal), visualization (equal), writing – review and editing (equal). **Marta Ezeta Watts:** investigation (equal), writing – review and editing (equal). **Megan K. B. Nolan:** investigation (equal), writing – review and editing (equal). **Francesca Benzoni:** conceptualization (equal), formal analysis (equal), resources (equal), supervision (equal), visualization (equal), writing – review and editing (equal).

## Funding

This research was funded by King Abdullah University of Science and Technology.

## Conflicts of Interest

The authors declare no conflicts of interest.

## Supporting information


**Data S1:** Supporting Information.

## Figures and Tables

**FIGURE A1:**
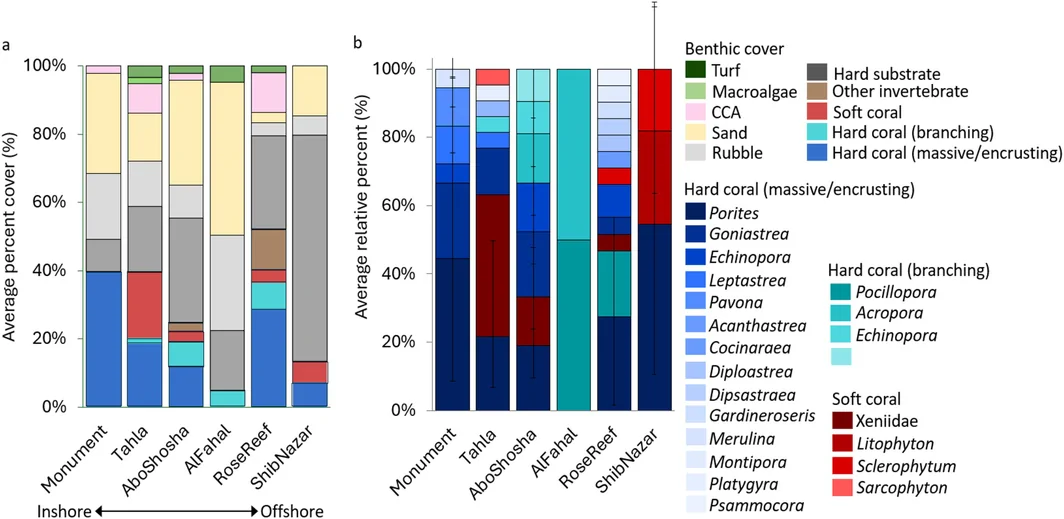
(a) Benthic cover and (b) coral community composition across the 6 study reefs in the central Red Sea at the start of the study period in Autumn 2022 (*T*_0_) as estimated by the point‐intercept method using orthomosaics.

**FIGURE A2:**
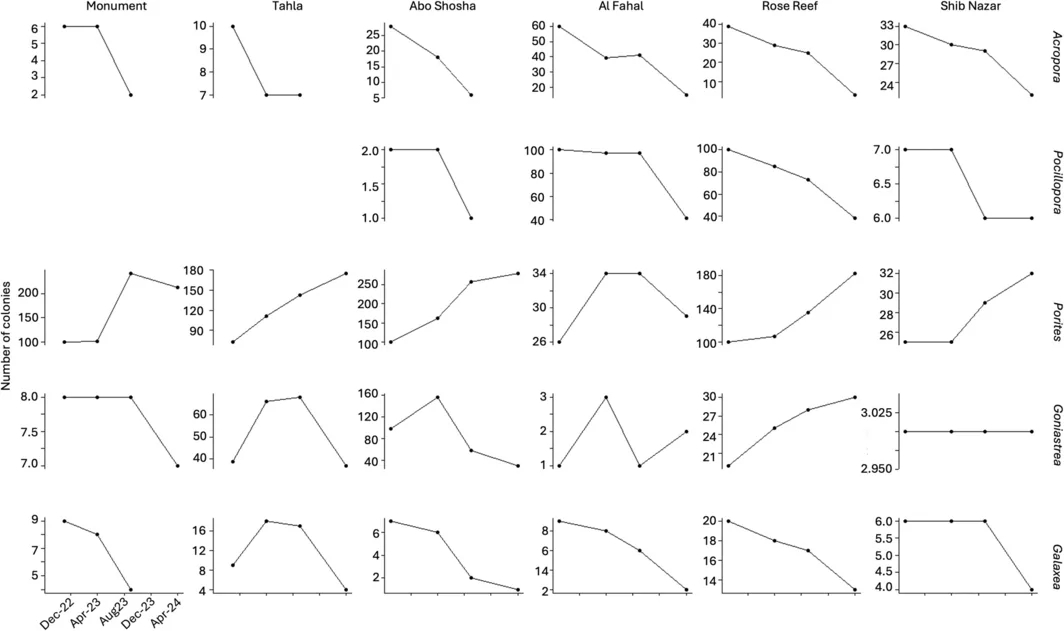
Number of living coral colonies that were tracked per reef and scleractinian taxon at each time point from September 2022 to April 2024 in the central Saudi Arabian Red Sea.

**FIGURE A3:**
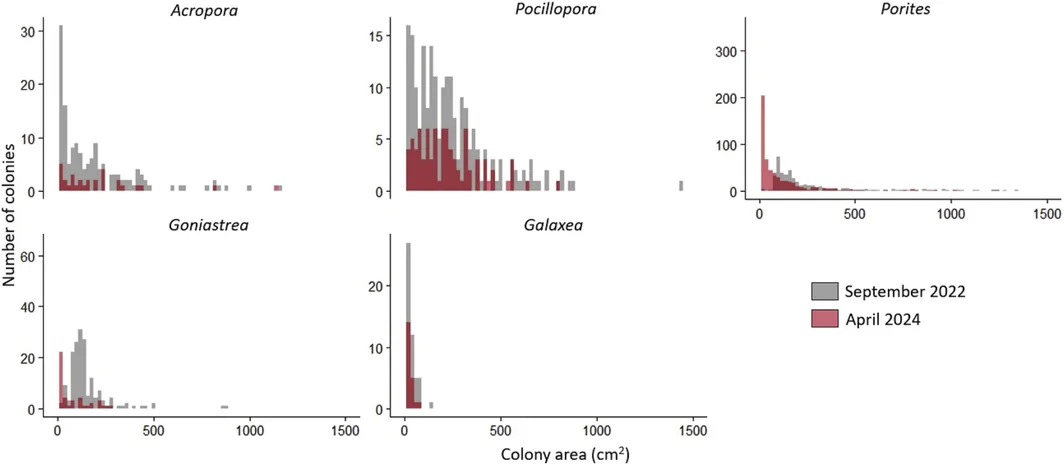
Colony size frequency of tracked coral colonies for each examined scleractinian taxon in September 2022 and April 2024 across the 6 study reefs in the central Saudi Arabian Red Sea.

**FIGURE A4:**
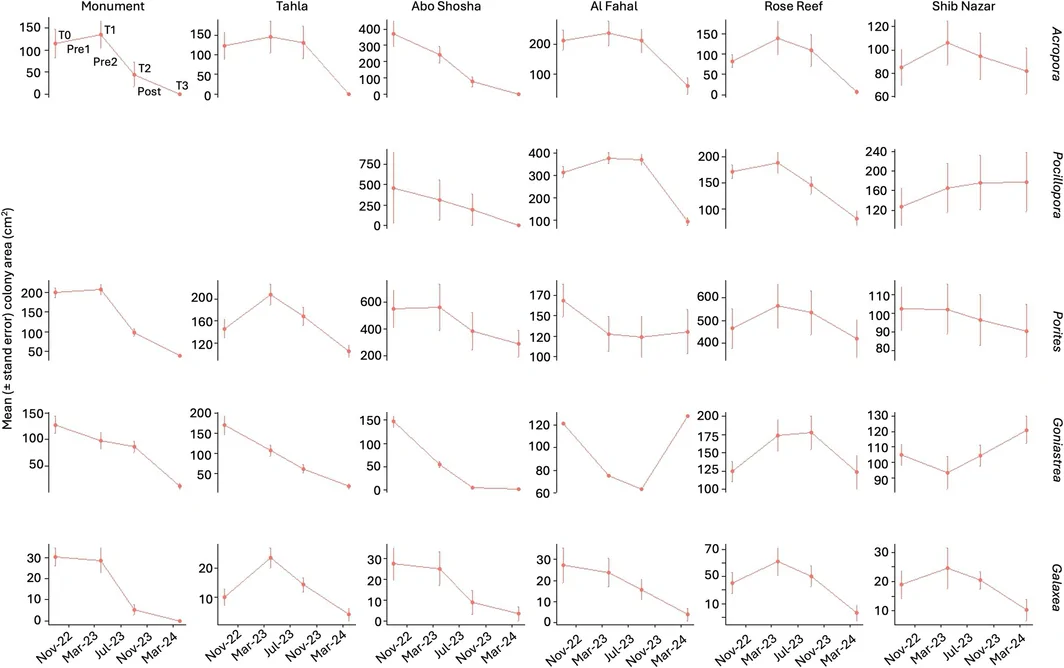
Average (± standard error) total coral colony area of each scleractinian taxon across the 6 study sites in the central Saudi Arabian Red Sea for each time point from September 2022 to April 2024 (*T*_0_–*T*_3_). The coral bleaching event occurred in September 2023 (*T*_2_). Time periods (Pre1, Pre2, and Post) between consecutive time points are indicated, and the total number of colonies tracked at each time point is plotted in Figure A2.

**FIGURE A5:**
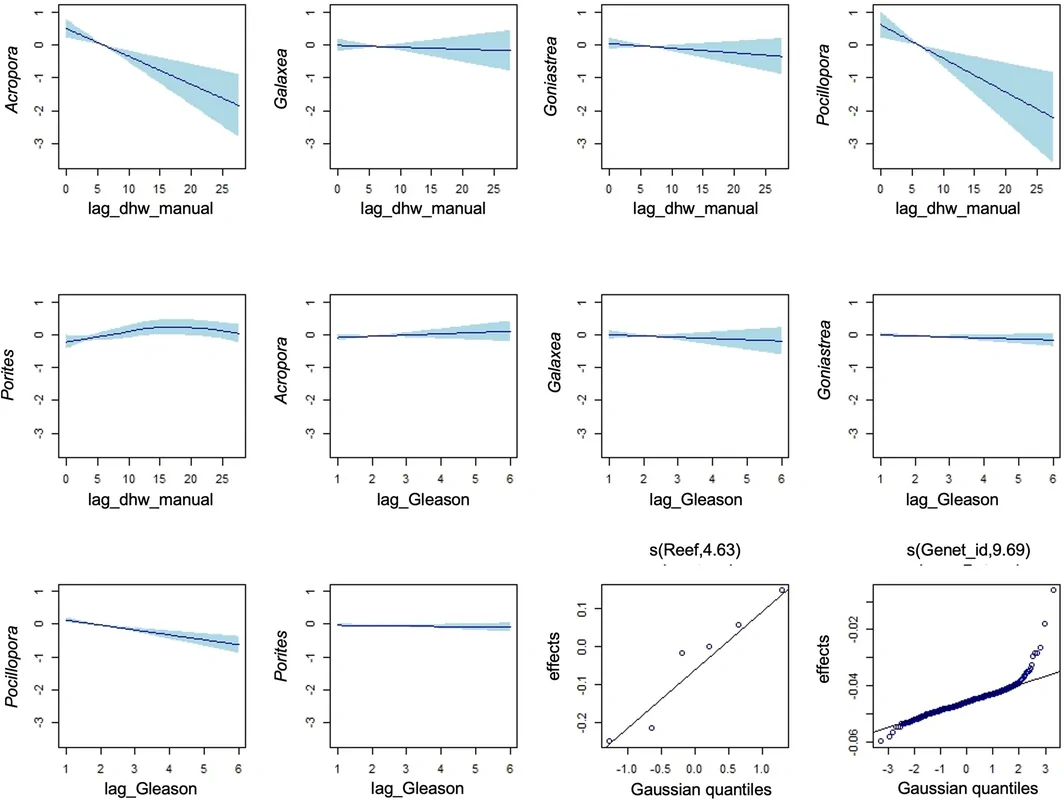
Partial effect plots of the generalized additive model modeling proportional change in coral colony area as a function of degree heating weeks, bleaching ranking, reef, scleractinian taxa, and colony for the 6 study reefs in the central Saudi Arabian Red Sea from September 2022 to April 2024. Pairwise comparisons and all *p*‐values are reported in Table A2.

**FIGURE A6:**
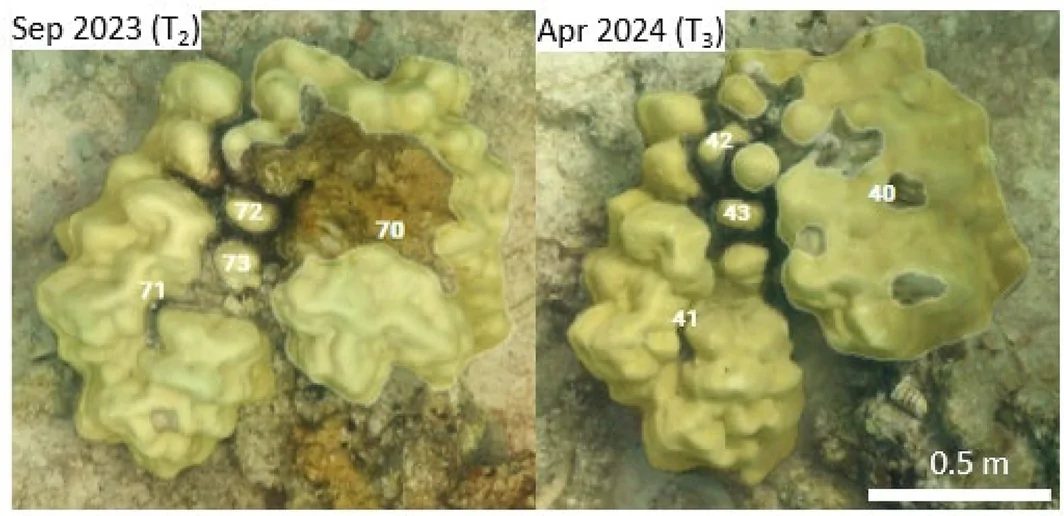
Example of tissue re‐growth over previously dead corallum surface of large *Porites* colony on Al Fahal (midshelf) in the central Red Sea from September 2023 to April 2024.

**FIGURE A7:**
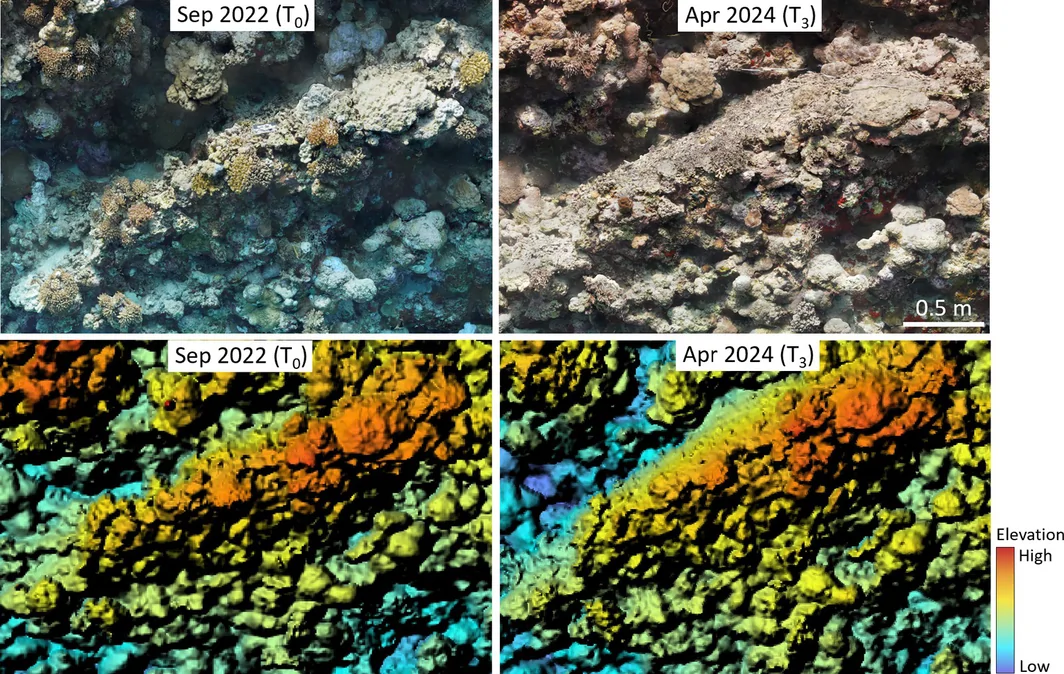
Example of rubble formation on Rose Reef (offshore) in the central Red Sea from September 2022 and April 2024, which could explain the increase in VRM on this reef shown on orthomosaics (top) and DEMs (bottom).

## Data Availability

Data used in this study are available in the Supporting Information.

## Appendix 1

**TABLE A1:** Output of the generalized additive model predicting probability of coral bleaching as a function of degree heating weeks, reef, and scleractinian taxa for the 6 study reefs in the central Saudi Arabian Red Sea from September 2022 to April 2024.

Parametric coefficients	Estimate	Std. Error	*z*	Pr(>|*z*|)	
(Intercept)	−4.3318	0.3709	−11.678	< 2e‐16	***
TaxaAcropora	1.285	0.3627	3.543	0.000396	***
TaxaGalaxea	1.9596	0.4009	4.888	1.02E‐06	***
TaxaGoniastrea	1.3785	0.5512	2.501	0.012382	*
TaxaPocillopora	2.3416	0.9292	2.52	0.011733	*
ReefAlFahal	0.1556	0.3126	0.498	0.618543	
ReefMonument	1.6167	0.8435	1.917	0.055294	.
ReefRoseReef	2.8514	0.3119	9.143	< 2e‐16	***
ReefShibNazar	2.3396	0.4002	5.846	5.03E‐09	***
ReefTahla	2.1072	0.4994	4.219	2.45E‐05	***

Approximate significance of smooth terms	edf	Ref.df	Chi.sq	*p*	
s(dhw_manual):TaxaPorites	4.355	5.389	195.812	< 2e‐16	***
s(dhw_manual):TaxaAcropora	1	1	79.227	< 2e‐16	***
s(dhw_manual):TaxaGalaxea	1.197	1.364	67.765	< 2e‐16	***
s(dhw_manual):TaxaGoniastrea	2.374	2.919	71.04	< 2e‐16	***
s(dhw_manual):TaxaPocillopora	2.734	3.231	173.119	< 2e‐16	***
s(Genet_Id)	7.428	1038	7.512	0.4	
R‐sq.(adj) = 0.587 Deviance explained = 54.1%					
‐REML = 717.19 Scale est. = 1 *n* = 2662					

	Contrast	Odds.ratio	SE	df	Null	t.ratio	*p*
Taxa	Porites/Acropora	1.687	0.491	2633	1	1.797	0.3756
	Porites/Galaxea	0.748	0.307	2633	1	−0.707	0.9549
	Porites/Goniastrea	9.05	6.29	2633	1	3.167	0.0135
	Porites/Pocillopora	6.052	2.47	2633	1	4.402	0.0001
	Acropora/Galaxea	0.444	0.161	2633	1	−2.234	0.1674
	Acropora/Goniastrea	5.365	3.66	2633	1	2.465	0.099
	Acropora/Pocillopora	3.587	1.33	2633	1	3.439	0.0054
	Galaxea/Goniastrea	12.095	8.91	2633	1	3.383	0.0065
	Galaxea/Pocillopora	8.088	3.78	2633	1	4.47	0.0001
	Goniastrea/Pocillopora	0.669	0.49	2633	1	−0.55	0.982
Reef	AboShosha/AlFahal	0.8559	0.268	2633	1	−0.498	0.9963
	AboShosha/Monument	0.1986	0.167	2633	1	−1.917	0.3921
	AboShosha/RoseReef	0.0578	0.018	2633	1	−9.143	< 0.0001
	AboShosha/ShibNazar	0.0964	0.0386	2633	1	−5.846	< 0.0001
	AboShosha/Tahla	0.1216	0.0607	2633	1	−4.219	0.0004
	AlFahal/Monument	0.232	0.191	2633	1	−1.777	0.481
	AlFahal/RoseReef	0.0675	0.0172	2633	1	−10.563	< 0.0001
	AlFahal/ShibNazar	0.1126	0.0355	2633	1	−6.92	< 0.0001
	AlFahal/Tahla	0.1421	0.0723	2633	1	−3.834	0.0018
	Monument/RoseReef	0.2909	0.231	2633	1	−1.553	0.6298
	Monument/ShibNazar	0.4853	0.396	2633	1	−0.887	0.9498
	Monument/Tahla	0.6123	0.487	2633	1	−0.617	0.9898
	RoseReef/ShibNazar	1.6684	0.509	2633	1	1.676	0.5476
	RoseReef/Tahla	2.1049	1	2633	1	1.564	0.6222
	ShibNazar/Tahla	1.2616	0.657	2633	1	0.447	0.9978

*Note:* Pairwise comparisons for taxa and all *p*‐values are reported.

**TABLE A2:** Output of the generalized additive model predicting change in proportional coral colony area as a function of degree heating weeks, bleaching ranking, reef, scleractinian taxa, and colony for the 6 study reefs in the central Saudi Arabian Red Sea from September 2022 to April 2024.

Parametric coefficients	Estimate	Std. Error	*t*	Pr(>|*t*|)	
(Intercept)	−0.04575	0.13274	−0.345	0.73037	
TaxaGalaxea	−0.59044	0.2023	−2.919	0.00355	**
TaxaGoniastrea	−0.24945	0.15851	−1.574	0.1157	
TaxaPocillopora	−0.03965	0.16864	−0.235	0.81415	
TaxaPorites	−0.40683	0.1467	−2.773	0.0056	**
Timeperiodpre1	0.63428	0.13249	4.787	1.81E‐06	***
Timeperiodpre2	−0.43055	0.21355	−2.016	0.04392	*
TaxaGalaxea:Timeperiodpre1	0.17058	0.23644	0.721	0.47071	
TaxaGoniastrea:Timeperiodpre1	−0.67562	0.15712	−4.3	1.79E‐05	***
TaxaPocillopora:Timeperiodpre1	−0.23977	0.17679	−1.356	0.17517	
TaxaPorites:Timeperiodpre1	−0.35599	0.14676	−2.426	0.01537	*
TaxaGalaxea:Timeperiodpre2	0.6655	0.32901	2.023	0.04323	*
TaxaGoniastrea:Timeperiodpre2	0.09985	0.27554	0.362	0.7171	
TaxaPocillopora:Timeperiodpre2	−0.52338	0.37374	−1.4	0.16156	
TaxaPorites:Timeperiodpre2	0.81022	0.27535	2.942	0.00329	**

Approximate significance of smooth terms	edf	Ref.df	*F*	*p*	
s(lag_dhw_manual):TaxaAcropora	1	1	14.508	0.000144	***
s(lag_dhw_manual):TaxaGalaxea	1	1	0.165	0.684395	
s(lag_dhw_manual):TaxaGoniastrea	1	1	1.191	0.275222	
s(lag_dhw_manual):TaxaPocillopora	1	1	10.204	0.001423	**
s(lag_dhw_manual):TaxaPorites	2.474	3.084	3.155	0.031021	*
s(lag_Gleason):TaxaAcropora	1	1	0.943	0.331684	
s(lag_Gleason):TaxaGalaxea	1	1	0.509	0.475819	
s(lag_Gleason):TaxaGoniastrea	1	1	1.467	0.22599	
s(lag_Gleason):TaxaPocillopora	1	1	21.228	4.43E‐06	***
s(lag_Gleason):TaxaPorites	1	1	0.506	0.477225	
s(Reef)	4.628	5	13.857	< 2e‐16	***
s(Genet_Id)	9.694	986	0.01	0.418696	
R‐sq.(adj) = 0.273 Deviance explained = 28.7%					
‐ML = 1637.7 Scale est. = 0.2858 *n* = 2057					

Contrast	Estimate	SE	df	t.ratio	*p*
*Acropora*					
post–pre1	−0.6343	0.132	2016	−4.787	< 0.0001
post–pre2	0.4305	0.214	2016	2.016	0.1086
pre1–pre2	1.0648	0.189	2016	5.635	< 0.0001
*Galaxea*					
post–pre1	−0.8049	0.197	2016	−4.083	0.0001
post–pre2	−0.235	0.265	2016	−0.888	0.6478
pre1–pre2	0.5699	0.186	2016	3.061	0.0063
*Goniastrea*					
post–pre1	0.0413	0.0865	2016	0.478	0.8816
post–pre2	0.3307	0.184	2016	1.801	0.1696
pre1–pre2	0.2894	0.161	2016	1.793	0.1723
*Pocillopora*					
post–pre1	−0.3945	0.121	2016	−3.255	0.0033
post–pre2	0.9539	0.332	2016	2.87	0.0116
pre1–pre2	1.3484	0.274	2016	4.928	< 0.0001
*Porites*					
post–pre1	−0.2783	0.0656	2016	−4.245	0.0001
post–pre2	−0.3797	0.214	2016	−1.778	0.1773
pre1–pre2	−0.1014	0.201	2016	−0.504	0.8693

*Note:* Pairwise comparisons and all *p*‐values are reported.

**TABLE A3:** Summary of the principal component analysis for changes in coral colony area for four of the target scleractinian taxa and three metrics of structural complexity across the 6 study reefs in the central Red Sea from September 2022 to April 2024.

	PC1	PC2
*Acropora*	0.17930	**0.60591**
*Pocillopora*	−0.24341	**0.55014**
*Goniastrea*	**0.58660**	0.01336
*Porites*	**0.38620**	−0.20866
Slope	**0.55457**	0.17599
VRM	0.29418	0.22566
Profile curvature	−0.14582	**0.45234**
Variance explained	34.80	23.40

*Note:* Values indicate loadings for each variable on the first two PCs, with the strongest loadings in bold.

**TABLE A4:** Output of the generalized additive model predicting probability of coral bleaching as a function of degree heating weeks, reef, and scleractinian taxa with transect as a random factor for the 6 study reefs in the central Saudi Arabian Red Sea from September 2022 to April 2024.

Parametric coefficients
Estimate	Std. Error	*z*	Pr(>|*z*|)	
(Intercept)	−4.3363	0.4611	−9.404	< 2e‐16	***
TaxaAcropora	1.3761	0.3729	3.691	0.000224	***
TaxaGalaxea	2.0457	0.4057	5.042	4.61E‐07	***
TaxaGoniastrea	1.3489	0.5189	2.6	0.009333	**
TaxaPocillopora	2.2245	0.8511	2.614	0.008961	**
ReefAlFahal	0.0525	0.4905	0.107	0.91476	
ReefMonument	1.6855	0.9157	1.841	0.065678	.
ReefRoseReef	2.9183	0.4891	5.967	2.42E‐09	***
ReefShibNazar	2.218	0.5521	4.017	5.89E‐05	***
ReefTahla	2.1113	0.6297	3.353	0.0008	***

Approximate significance of smooth terms	edf	Ref.df	Chi.sq	*p*	
s(dhw_manual):TaxaPorites	4.521	5.492	192.196	< 2e‐16	***
s(dhw_manual):TaxaAcropora	1.001	1.001	73.673	< 2e‐16	***
s(dhw_manual):TaxaGalaxea	1.215	1.395	66.014	< 2e‐16	***
s(dhw_manual):TaxaGoniastrea	2.221	2.726	63.568	< 2e‐16	***
s(dhw_manual):TaxaPocillopora	2.604	3.07	158.784	< 2e‐16	***
s(Genet_Id)	4.216	1038	4.245	0.433992	
s(Transect)	6.908	12	20.27	0.000944	***
R‐sq.(adj) = 0.591 Deviance explained = 54.8%					
‐REML = 712.79 Scale est. = 1 *n* = 2662					

	Contrast	Odds.ratio	SE	df	Null	*t*.ratio	*p*
Taxa	Porites/Acropora	2.14	0.692	2629	1	2.351	0.1293
	Porites/Galaxea	0.931	0.401	2629	1	−0.166	0.9998
	Porites/Goniastrea	9.652	6.53	2629	1	3.349	0.0074
	Porites/Pocillopora	7.054	2.98	2629	1	4.624	< 0.0001
	Acropora/Galaxea	0.435	0.158	2629	1	−2.289	0.1486
	Acropora/Goniastrea	4.511	2.96	2629	1	2.298	0.1457
	Acropora/Pocillopora	3.297	1.24	2629	1	3.182	0.0128
	Galaxea/Goniastrea	10.367	7.36	2629	1	3.292	0.0089
	Galaxea/Pocillopora	7.577	3.56	2629	1	4.311	0.0002
	Goniastrea/Pocillopora	0.731	0.515	2629	1	−0.445	0.9919
Reef	AboShosha/AlFahal	0.9489	0.465	2629	1	−0.107	1
	AboShosha/Monument	0.1853	0.17	2629	1	−1.841	0.4397
	AboShosha/RoseReef	0.054	0.0264	2629	1	−5.967	< 0.0001
	AboShosha/ShibNazar	0.1088	0.0601	2629	1	−4.017	0.0009
	AboShosha/Tahla	0.1211	0.0762	2629	1	−3.353	0.0105
	AlFahal/Monument	0.1953	0.175	2629	1	−1.828	0.4479
	AlFahal/RoseReef	0.0569	0.0257	2629	1	−6.338	< 0.0001
	AlFahal/ShibNazar	0.1147	0.0562	2629	1	−4.42	0.0001
	AlFahal/Tahla	0.1276	0.081	2629	1	−3.243	0.0152
	Monument/RoseReef	0.2915	0.252	2629	1	−1.428	0.7102
	Monument/ShibNazar	0.5871	0.521	2629	1	−0.601	0.991
	Monument/Tahla	0.6533	0.574	2629	1	−0.484	0.9967
	RoseReef/ShibNazar	2.0143	0.973	2629	1	1.45	0.6962
	RoseReef/Tahla	2.2412	1.35	2629	1	1.341	0.7622
	ShibNazar/Tahla	1.1127	0.715	2629	1	0.166	1

*Note:* Pairwise comparisons for taxa and all *p*‐values are reported.
